# *Rhus coriaria* increases protein ubiquitination, proteasomal degradation and triggers non-canonical Beclin-1-independent autophagy and apoptotic cell death in colon cancer cells

**DOI:** 10.1038/s41598-017-11202-3

**Published:** 2017-09-14

**Authors:** Khawlah Athamneh, Hussain El Hasasna, Halima Al Samri, Samir Attoub, Kholoud Arafat, Nehla Benhalilou, Asma Al Rashedi, Yusra Al Dhaheri, Synan AbuQamar, Ali Eid, Rabah Iratni

**Affiliations:** 1 Department of Biology, College of Science, United Arab Emirates University, Al-Ain, P.O. Box 15551 United Arab Emirates; 2 Department of Pharmacology & Therapeutics, College of Medicine & Health Sciences, United Arab Emirates University, Al-Ain, P.O. Box: 17666 United Arab Emirates; 3 Department of Pharmacology and Toxicology, Faculty of Medicine, American University of Beirut, Beirut, PO Box 11-0236 Lebanon

## Abstract

Colorectal cancer is the fourth leading cause of cancer-related deaths worldwide. Here, we investigated the anticancer effect of *Rhus coriaria* extract (RCE) on HT-29 and Caco-2 human colorectal cancer cells. We found that RCE significantly inhibited the viability and colony growth of colon cancer cells. Moreover, RCE induced Beclin-1-independent autophagy and subsequent caspase-7-dependent apoptosis. Blocking of autophagy by chloroquine significantly reduced RCE-induced cell death, while blocking of apoptosis had no effect on RCE-induced cell death. Mechanistically, RCE inactivated the AKT/mTOR pathway by promoting the proteasome-dependent degradation of both proteins. Strikingly, we also found that RCE targeted Beclin-1, p53 and procaspase-3 to degradation. Proteasome inhibition by MG-132 not only restored these proteins to level comparable to control cells, but also reduced RCE-induced cell death and blocked the activation of autophagy and apoptosis. The proteasomal degradation of mTOR, which occurred only 3 hours post-RCE treatment was concomitant with an overall increase in the level of ubiquitinated proteins and translated stimulation of proteolysis by the proteasome. Our findings demonstrate that *Rhus coriaria* possesses strong anti-colon cancer activity through stimulation of proteolysis as well as induction of autophagic and apoptotic cell death, making it a potential and valuable source of novel therapeutic cancer drug.

## Introduction

Cancer therapies have witnessed great advances in the recent past; however, cancer continues to be a leading cause of death, with colorectal cancer being the fourth cause of cancer-related deaths^1^. Colorectal cancer affects both sexes equally with poor survival rate once it metastasizes^1^.

Phytochemicals, which are plant derived compounds that have been increasingly utilized as anti-cancer drugs due to accumulated evidences that support their potential^2^. Therefore, phytochemicals gained a vital role in the area of experimental cancer research, because they are effective and often with less side effects. Examples of anti-cancer drugs that have been derived from plants and are currently in clinical use include Taxol (isolated from *Taxus brevifolia* Nutt) and the DNA topoisomerase I inhibitor camptothecin (isolated from *Camptotheca acuminate*)^3, 4^.

*Rhus coriaria*, more commonly known as sumac, belongs to the Anacardiaceae family. It originates from the Mediterranean countries^5^. In addition to its use as a condiment, this plant has also been used in herbal and traditional medicine for hundreds of years. Moreover, *R*. *coriaria* has attracted more attention in the recent past due to its therapeutic values^6^. Indeed, accumulated evidence shows that this plant is rich in phytochemical compounds such as tannins, phenolic acids, flavonoids, and organic acids^7^. Furthermore, recent, studies have shown that sumac possesses potent antioxidant activities, likely due to its phenolic compounds^8^. Added to that, Rhus coriaria was shown to possess therapeutic properties for many diseases, such as type II diabetes^9^, osteoarthritis^10^, and cardiovascular diseases^11^.

In addition to *R*. *coriaria*’s antioxidant activities, it holds other therapeutic values, for example it has antifungal^12^ and antibacterial activities, where it has an effect against gram positive and negative bacteria^13^.

Recently, we showed for the first time, that RCE possesses anti-cancer effects against human triple-negative breast cancer cells. We reported that RCE significantly inhibited proliferation, adhesion, migration and invasion of MDA-MB 231 cells as well as suppressed angiogenesis and tumor growth *in ovo*. Interestingly, the same concentrations utilized for the MDA-MB231 cells were found to have no cytotoxic effects on Human Umbilical Vein Endothelial Cells (HUVECs). Moreover, we also reported that RCE induced senescence and cell cycle arrest at G1 phase, as well as autophagy. This autophagy appears to be the main mechanism of RCE-induced cell death^14, 15^.

Autophagy is a highly regulated cellular process that can either result in the degradation of proteins or it can specifically target distinct organelles. For example, mitochondria in mitophagy or the endoplasmic reticulum in reticulophagy are some forms of autophagy that helps rid the cell of damaged or expired organelles and proteins^16^. Initiation of autophagy starts with the engulfment of damaged or unnecessary cellular content into a double-membrane vesicle (autophagosome), which is then transported to and fused with the lysosome to form a single-membrane autolysosome, the material in which would be ultimately degraded and recycled^17^. Fundamentally, autophagy is a cellular survival mechanism, where it mediates the turnover of protein aggregates that might otherwise lead to cellular dysfunction. Nevertheless, if the intracellular damage or organellular expiry is exceedingly high, autophagy becomes a “killing” rather than a “rescuing” mechanism. This is due to the fact that large proportions of the cytoplasm would be destroyed resulting in an irreversible cellular atrophy, and a consequential collapse of crucial cellular functions^18, 19^.

The ubiquitin proteasome system (UPS) is a highly regulated and extremely selective cellular mechanism of protein degradation. The degradation of most nuclear and cytosolic proteins is the responsibility of the UPS, including misfolded proteins, short-lived as well as long-lived proteins. Proteins are marked for degradation by the attachment of a ubiquitin (Ub) chain to them^20, 21^. The initiation of the cascade of events leading up to protein degradation begins with the activation of ubiquitin by Ub-activating enzyme (E1), followed immediately by the delivery of ubiquitin to Ub-conjugating enzyme (E2). Then, ligation of the polyubiquitin chain to the substrate via Ub-ligase enzyme (E3). Finally, the proteasomes recognize the polyubiquitin chain as a target for degradation and destroys the substrate^22, 23^. Thus, sealing the fate of the protein to damnation.

Until recently, the UPS and autophagy were looked at as independent pathways serving distinct functions. However, recent studies revealed the existence of an interaction between the UPS and autophagy, suggesting a coordinated and complementary relationship between these degradation systems during cellular stresses^24^. Indeed, the two mechanisms of protein degradation often coexist to deal with cellular proteotoxic stress^25^.

Interestingly, the finding that proteasome overload or inhibition induces autophagy in many cell types provided a strong indication for a functional link between the UPS and autophagy^26^. It seems that the proteasome-to-autophagy direction of regulation primarily compensates the exceeded degradative capacity of the UPS and eliminates the threat resulting from damaged organelles or the accumulation of potentially toxic protein aggregates^26^. Recently, the anti-cancer drug and proteasome inhibitor bortezomib was found to trigger compensatory autophagy^27^. Hence, autophagy might act as a backup mechanism that can help to relieve the burden of the overloaded UPS^28^. Conversely, an activation of the proteasome upon impairment of autophagy seems unlikely and lacks strong evidences.

The UPS and autophagy seem to share some regulatory proteins that might be a key player in mediating the cross-talk between these two mechanisms. This includes p62/SQSTM1^24^ and the histone deacetylase 6 (HDAC6)^29^. p62 seems to redirect ubiquitinated proteasomal proteins into the autophagosome upon proteasome inhibition or overload. Still, the exact mechanism through which this change of destination occurs remain unknown^24, 30^. HDAC6 represents another important factor in the communication between autophagy and the proteasome^29^. Pandey, *et al*. showed in *Drosophila* that autophagy was activated to compensate for UPS impairment in a histone deacetylase 6- (HDAC6) dependent manner^29^. Moreover, HDAC6 overexpression rescued UPS impairment in an autophagy dependent fashion^29^. A subsequent study indicates that that HDAC6 promotes autophagosome-lysosome fusion in ubiquitin-mediated selective quality control autophagy^31^. Thus, ubiquitin seems to represent the common denominator shared by the UPS and autophagy under the umbrella of a single proteolysis network^27^.

Although the functional relationship between the UPS and autophagy is becoming more evident nowadays, the exact molecular mechanism(s) through which the function of these two degradation systems is coordinated remain largely obscure^25^. Understanding of the molecular mechanism through which the autophagy and UPS cross-talk in response to different stresses will be useful for therapeutic goals and will certainly contribute to the development on novel therapies for various diseases including cancer.

In the present study, we investigated the cytotoxic effects of *R*. *coriaria* extract against human colon cancer cells. Our results demonstrate that *R*. *coriaria* exerts its anti-colon cancer effect at least partly through inactivation of mTOR, concomitant with stimulation of the global protein ubiquitination and the ubiquitin proteasome system. This early event serves as a trigger for the induction of non-canonical autophagy and subsequent caspase-7-dependent apoptosis, which together ultimately lead to cellular death of colon cancer cells.

## Results

### Inhibition of cellular viability of human HT-29 and Caco-2 colon cancer cells by *Rhus coriaria* extract

**Figure 1:**
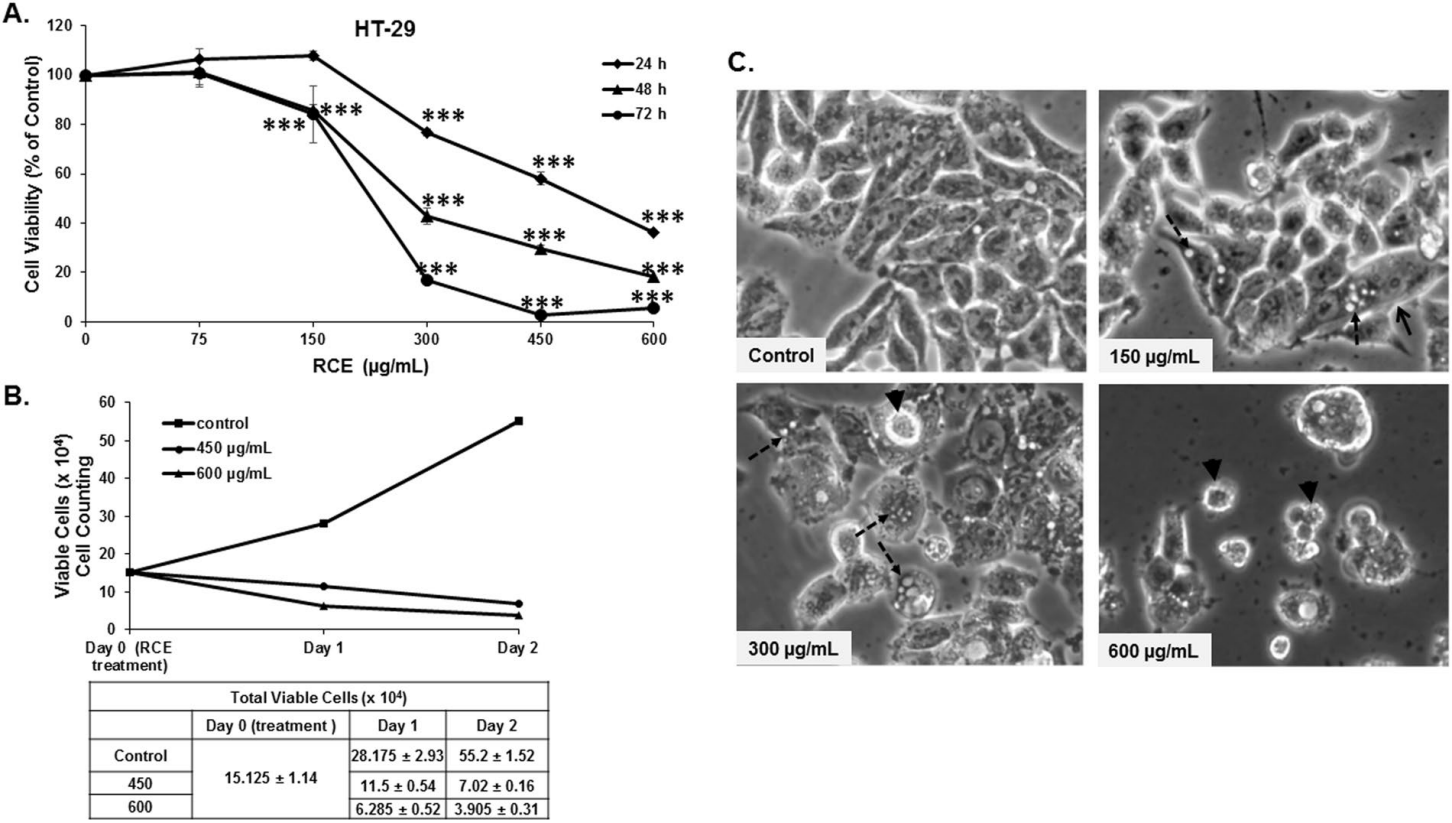
Inhibition of cellular viability by *Rhus coriaria*. (**A**) Exponentially growing HT-29 colon cancer cells were treated with and without the indicated concentrations of RCE for 24, 48 and 72 h. Viability was measured as described in Materials and Methods. Data represent the mean of six independent experiments carried out in triplicate. Statistical analysis for cell viability data on control or treated cells were performed using one-way ANOVA followed by LSD Post-Hoc test to determine the significance (***p < 0.001). (**B**) Determination of cellular viability through cell counting. HT-29 cells were exposed to RCE for 24 h and 48 and cell viability was monitored using the Muse cell analyzer as described in materials and methods. Data represent the mean ± SEM of three independent experiments. (**C**) Morphological changes in RCE-treated HT-29 cells. Morphological changes observed in the treated HT-29 cells after 48 h of treatment with the indicated concentration of RCE. Cells were observed under EVOS XL Core Cell Imaging System (Life Technologies) at 400×.

To examine the anticancer activity of RCE on human colon cancer, we measured the effect of increasing concentrations of the RCE (0, 75, 150, 300, 450 and 600 µg/mL) on the proliferation of HT-29 (Fig. 1A) and Caco-2 (Figure S1A) human colon cancer cell lines using an assay based on monitoring of cell metabolic activity. Our results showed that exposure of HT-29 or Caco-2 cells to RCE decreased cellular viability in a concentration and time-dependent manner. For the HT-29 cells, the IC_50_ values at 24, 48 and 72 hours are 518, 346 and 271 µg/mL, respectively. As for Caco-2 cells, IC_50_ at 24 and 48 hours are 384 and 316 µg/mL, respectively. It is noteworthy to mention that *Rhus coriaria* had no effect on cellular viability of the normal human epithelial mammary cells (HMECs) (data not shown).

Cell viability was also monitored using an assay which differentially stains viable and dead cells based on their permeability to two DNA binding dyes as described in the materials and methods section. We found that RCE treatment also led to a time- and concentration-dependent decline in the number of viable HT-29 cells, indicative of cell death, when compared to the number of cells counted at the day of treatment (day 0) (Fig. 1B).

Light microscopy observation of HT-29 (Fig. 1C) and Caco-2 (Figure S1B) cells upon RCE treatment revealed several morphological changes in both cell lines compared to control cells. Indeed, a subpopulation of RCE-treated cells revealed cytoplasmic vacuolation (dashed arrows). At higher concentrations of RCE (600 µg/mL), a subpopulation of cells appeared smaller and rounded, characteristic of dying cells (arrowheads).

### *Rhus coriaria* inhibits HT-29 colony growth

**Figure 2:**
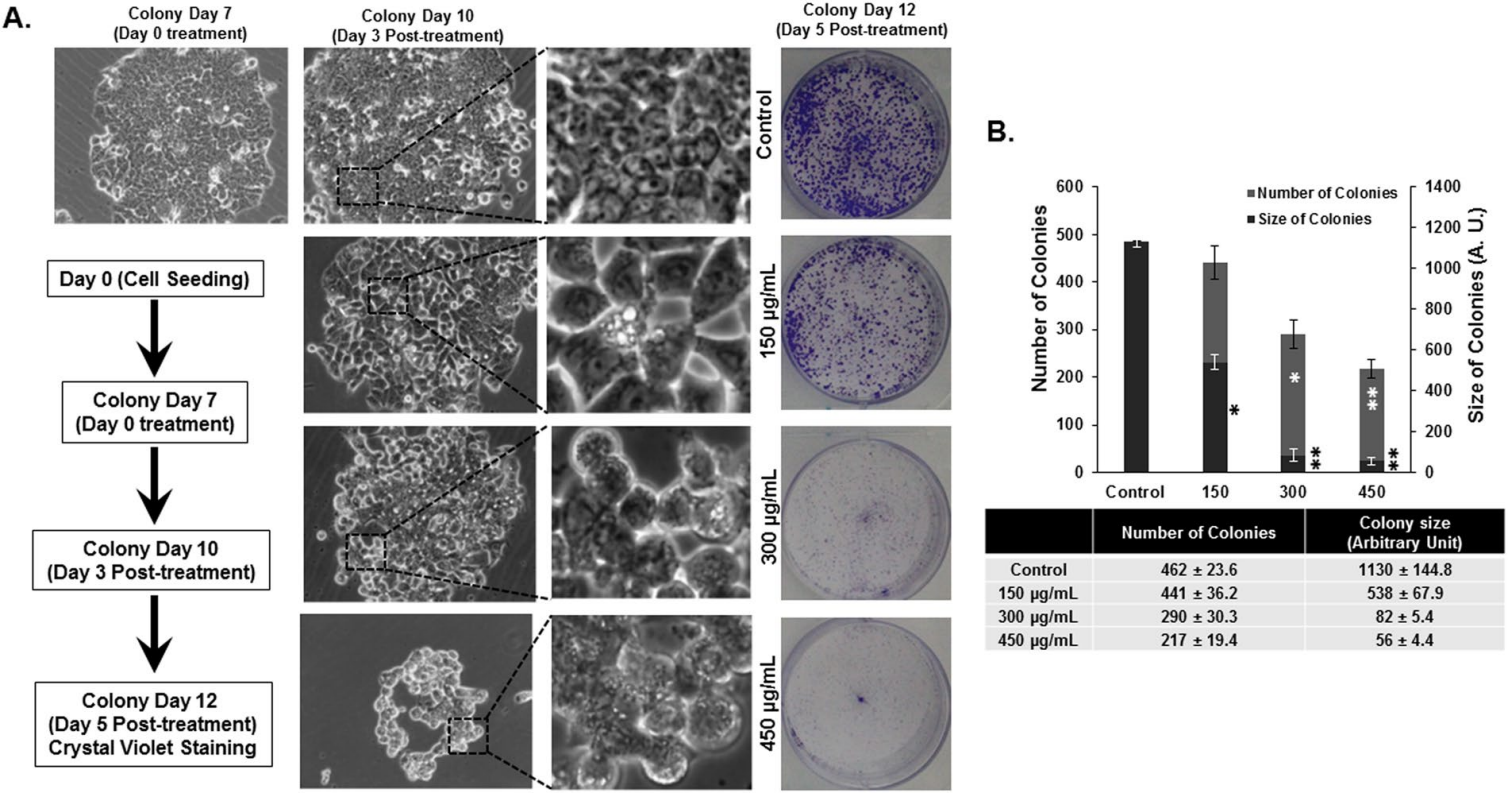
*Rhus coriraria* inhibits HT-29 colony growth. (**A**) HT-29 colonies were first allowed to form in normal media for seven days as described in Materials and Methods. Formed colonies were then treated with or without increasing concentrations of RCE and allowed to grow for five more days before crystal violet staining. Size and morphology of the growing colonies was followed over time under EVOS XL Core Cell Imaging System (Life Technologies) at 40×. (**B**) Inhibition of colony growth was assessed by measuring the number and size (surface area) of the colonies obtained in control and RCE-treated plate as described in Materials and methods. Data represent the mean of three independent experiments carried out in triplicate. Statistical analysis for cell viability data on control or treated cells were performed using one-way ANOVA followed by LSD Post-Hoc test to determine the significance (*p < 0.05, **p < 0.005).

To further confirm the anticancer potential of *Rhus coriaria*, we sought to determine if RCE modulates proliferative capacity of HT-29 colonies formed in culture. To this end, HT-29 cells were grown for seven days to form visible colonies and then treated for five days with various concentrations of RCE. As shown in Fig. 2A and B, treatment with RCE caused a concentration-dependent significant decrease in the number and size of colonies. This significant reduction in number and size of colonies is clearly indicative of massive cell death. In addition, microscopic observation of the treated colonies shows cellular vacuolation suggesting the induction of autophagy. Colony formation by HT-29 was also assessed using the soft agar colony formation assay as described in supplementary methods section. As shown in Supplementary Figure S2A,B, RCE markedly reduced the number of colonies when grown on soft agar. Taken together, these data further confirm the growth inhibitory effect of RCE on the proliferation of HT-29 cells.

### Induction of non-canonical Beclin-1 independent autophagy in colon cancer cells by *Rhus coriaria*

Morphological observation of RCE-treated HT-29 and Caco-2 colon cancer cells (Figs 1B and S1B, respectively) revealed massive cytoplasmic vacuolation indicative of possible induction of autophagy (dashed arrows). To confirm the autophagic origin of those vacuoles, we used a fluorescence marker of autophagy vacuoles as described in supplementary Methods section. As it is shown, in Figure S3, exposure of HT-29 cells for 24 h to RCE led to an accumulation of autophagic vacuoles, thus confirming the induction of autophagy by RCE in colon cancer cells.

**Figure 3:**
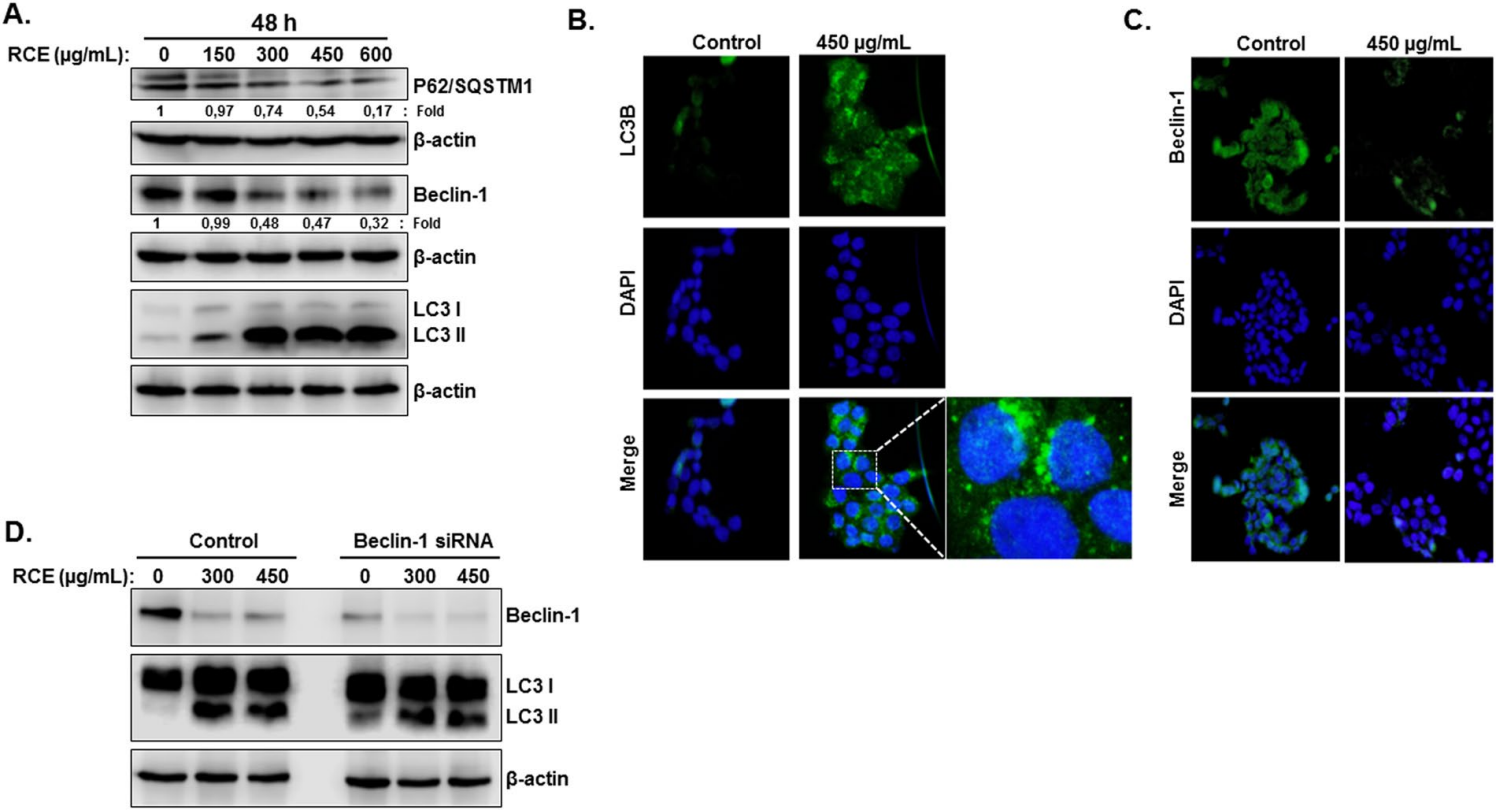
Induction of non-canonical Beclin-1 independent autophagy by *Rhus coriaria* in colon cancer cells. (**A**) Western blotting analysis of LC3-II, p62 (SQSTM1), and Beclin-1 expression in RCE-treated HT-29 cells. Cells were treated with or without increasing concentrations of RCE for 48 h, then whole cell proteins were extracted and subjected to Western blot analysis, as described in Materials and Methods, for LC3-II, 62 (SQSTM1), Beclin1 and β-actin (loading control) proteins. The western blots shown are representative of at least three independent experiments. (**B**) Immunofluorescence staining of LC3B in RCE-treated HT-29 cells. HT-29 cells were treated with RCE (450 µg/mL) for 24 h and then cells were stained with antibody specific for LC3B (cell signaling) and DAPI as described in Materials and Methods. (**C**) Immunofluorescence staining of Beclin-1 in RCE-treated HT-29 cells. HT-29 cells were treated with RCE (450 µg/mL) for 24 h and then cells were stained with antibody specific for Beclin-1 (cell signaling) and DAPI.

We next examined the protein expression of markers specific for autophagy. Autophagy is characterized by the conversion of LC3-I (cytosolic form) into a lipidized LC3-II (autophagosome membrane-bound form). We therefore, analyzed the accumulation of LC3-II by Western blotting. Figure 3A shows that RCE induced a concentration-dependent accumulation of the LC3-II starting at 300 µg/mL RCE in HT-29 Cells. Similarly, RCE also induced a concentration-dependent accumulation of the LC3-II in Caco-2 cells (Figure S4A). Moreover, immunofluorescence staining for endogenous LC3B revealed clear LC3-positive puncta in RCE treatment in HT-29 cells (Fig. 3B). Endogenous LC3B was hardly detectable in control cells (Fig. 3B). The expression of another widely used autophagy-specific marker p62(SQSTM1), a ubiquitin-binding protein involved in autophagy and whose level decreases when autophagy flux increases, was also evaluated. Figure 3A shows a decrease in p62(SQSTM1) level at 300 µg/mL, suggesting that autophagy is induced by a concentration ≥300 µg/mL RCE. Hence, the conversion of LC3-I/II, detection of LC3-positive puncta along with the downregulation of p62 (SQSTM1) confirm the formation of autophagosome in RCE-treated cells. Next, we assessed the expression of Beclin-1, the autophagy effector that plays a key role in autophagosome formation. Surprisingly, we found that Beclin-1 level in HT-29 cells decreased in a dose-dependent manner in response to RCE treatment starting at concentration of 300 µg/mL RCE (Fig. 3A). Similarly, Beclin-1 decrease was also observed in RCE-treated Caco-2 cells starting at 300 µg/mL RCE (Figure S4A).

Downregulation of Beclin-1 protein level was also confirmed by immunofluorescence staining of Beclin-1 in HT-29 cells cells treated with 450 µg/mL RCE (Fig. 3C). Altogether, our data confirms that RCE induces a Beclin-1 independent autophagy in colon cancer cells.

To confirm that Beclin-1 is not required for RCE-induced autophagy, we knocked down beclin-1 protein in HT-29 cells using Beclin-1-specific siRNA. As it is shown in Fig. 3D, Beclin-1 knockdown did not inhibit LC3-II accumulation and hence RCE-induced autophagy. Taken together, these data provide convincing evidence that exposure of colon cancer cells to *Rhus coriaria* triggers a Beclin-1 independent autophagy.

**Figure 4:**
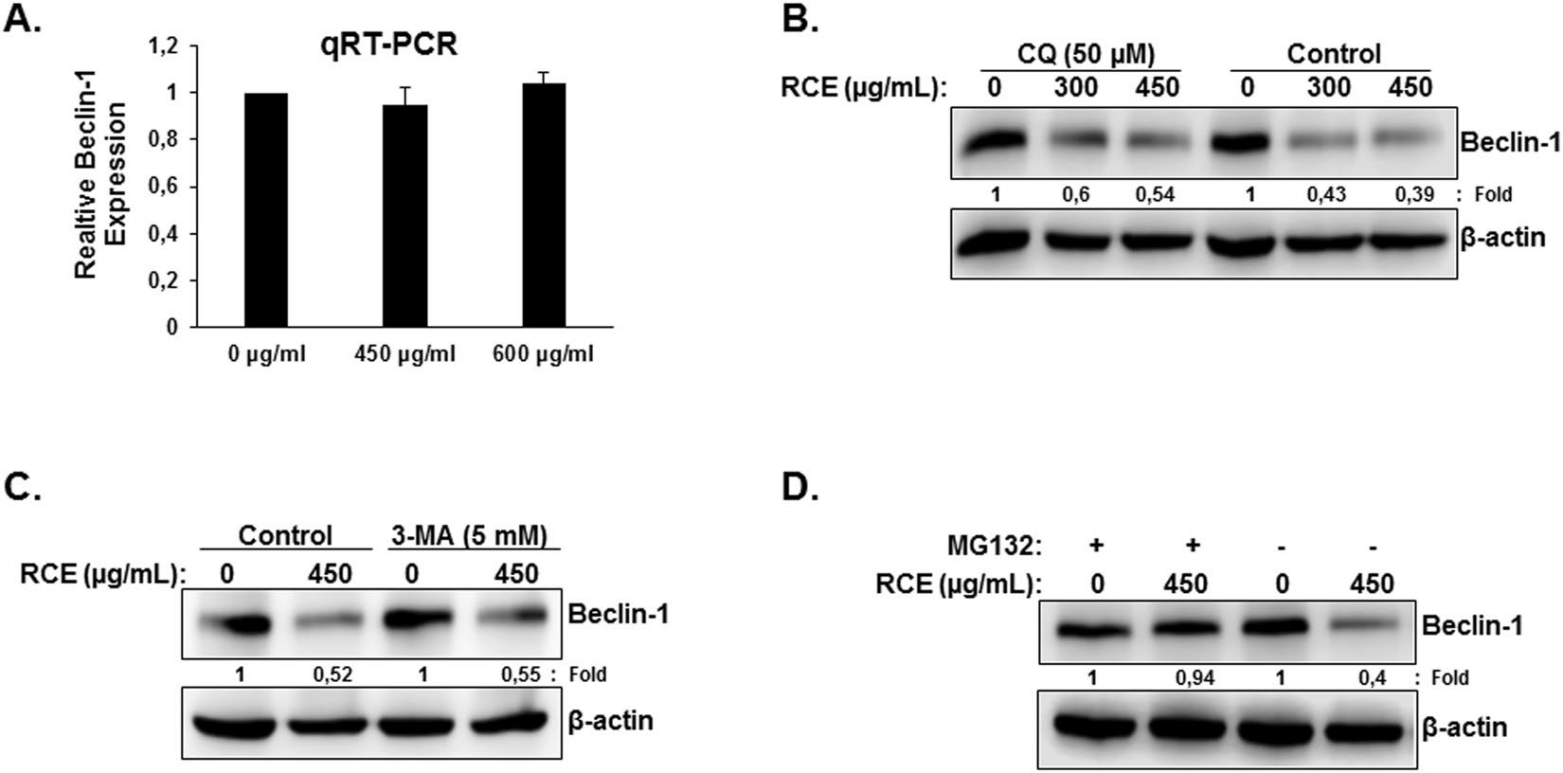
*Rhus coriaria* targets Beclin-1 to proteasome degradation in HT-29 cells. (**A**) RCE does not affect the levels of Beclin-1 transcripts. Total RNA from RCE-treated and untreated cells were used to amplify, by qRT-PCR, the Beclin-1 transcripts using Beclin-1 specific primers. GAPDH was used as internal normalization control. (**B**,**C**) RCE-mediated downregulation of Beclin-1 is autophagy-independent. Cells were pretreated with or without CQ (50 µM) (**B**) or 3-MA (**C**) for 1 h and then RCE was added at the indicated concentrations for 48 h. Proteins were extracted and Beclin-1 protein level was determined by western blot. (**D**) RCE targets Beclin-1 to proteasome degradation and inhibitors of the proteasome (MG-132) restore Beclin-1 protein levels. HT-29 cells were pre-treated for 2 h with or without MG-132 (15 µM) prior to treatment with RCE (450 µg/mL). Whole cell lysate were resolved on SDS-PAGE and analyzed for Beclin-1 protein.

To investigate the mechanism of Beclin-1 downregulation upon RCE treatment, we first examined the level of Beclin-1 transcript using qRT-PCR in HT-29 cells. As shown in Fig. 4A, the level of Beclin-1 mRNA remained unaffected by RCE treatment indicating that the downregulation of Beclin-1 is a posttranscriptional event. We tested whether Beclin-1 downregulation is a result of its autophagolysosomal degradation by inhibiting the autophagolysosome formation using the autophagy inhibitor chloroquine (CQ) and then measured the level of Beclin-1. As it is shown in Fig. 4B, blockade of autophagosome degradation by CQ failed to restore Beclin-1 protein levels in response to RCE. Inhibition of autophagosome formation by the autophagy inhibitor 3-methyl adenine (3-MA) also failed to restore Beclin-1 protein level (Fig. 4C), hence, suggesting that downregulation of the protein level of Beclin-1 is autophagy-independent. We next examined whether Beclin-1 is targeted for proteasome degradation upon RCE treatment. Toward this, cells were first pre-treated for 2 hours with the proteasome inhibitor MG-132 and then treated with RCE. Interestingly, we found that proteasome inhibition completely abrogated the RCE-induced decrease of Beclin-1, whose level remained comparable to control cells (Fig. 4D). This result clearly indicates that RCE targets Beclin-1 to proteasome degradation.

### *Rhus coriaria* induces caspase-7-dependent apoptosis in colon cancer cells

**Figure 5:**
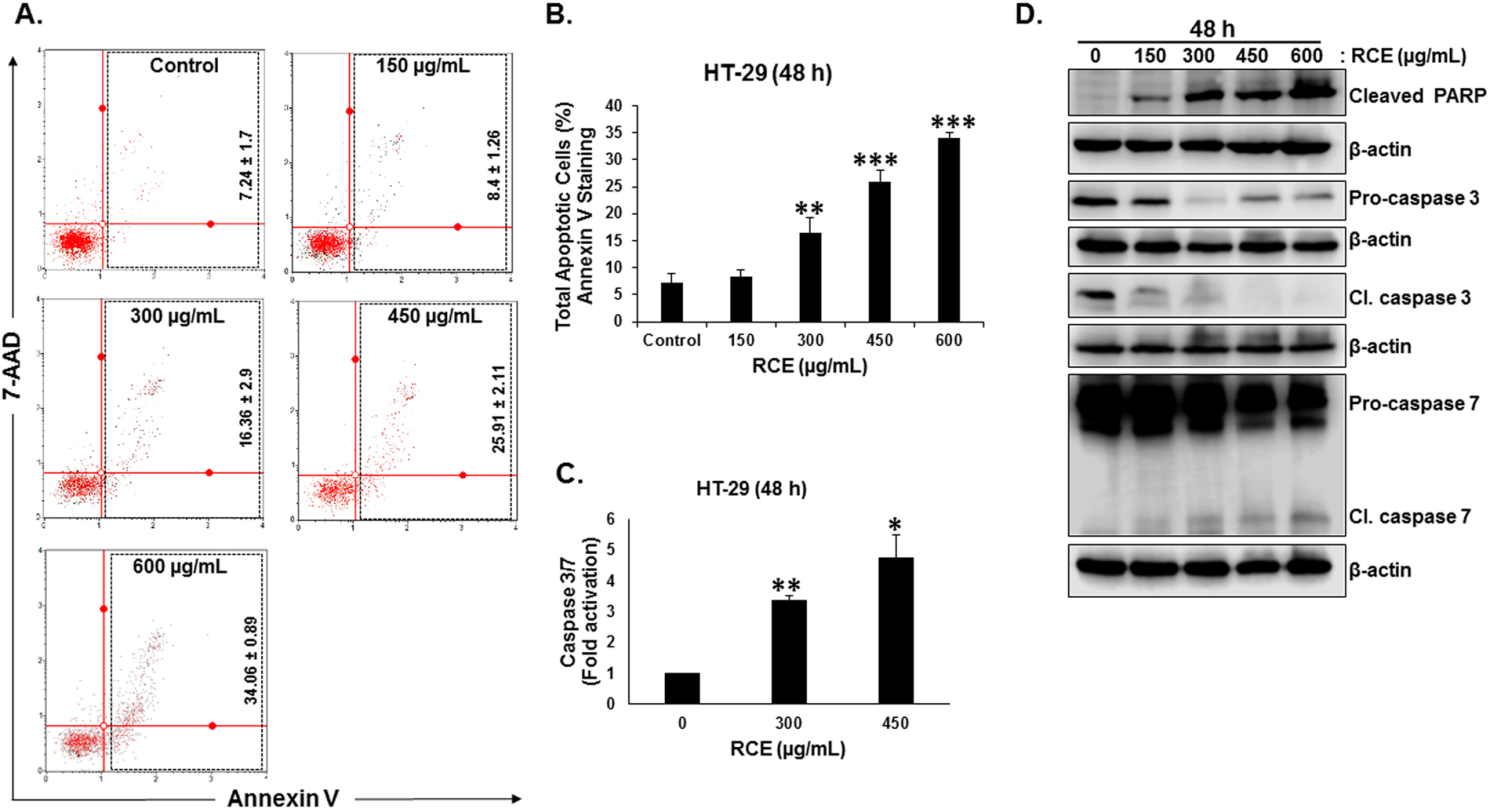
Induction of caspase-7-mediated apoptosis by *Rhus coriaria* in HT-29 cells. **(A**,**B)** RCE induced apoptosis in the HT-29 cells. Annexin V binding was carried out using Annexin V & Dead Cell kit (Millipore). Cells were treated with or without increasing concentrations of RCE for 48 h. Detached and adherent cells were collected and stained with Annexin V and 7-AAD and then the events for total apoptotic cells were counted with the Muse^TM^ Cell Analyzer as described in Materials and Methods. (**C**) Stimulation of caspase 3/7activity in HT-29 cells cells after exposure to RCE (300 and 450 µg/mL) for 48 h. The relative caspase 3/7 activity was normalized to the number of viable cells per well and is expressed as fold of activation compared to the control cells. (**D**) Western blot analysis of caspase-3, -7 activation and PARP cleavage in RCE-treated HT-29 cells after 48 h treatment. Data represent the mean ± SEM of at least 3 independent experiments. Statistical analysis for cell viability data on control or treated cells were performed using one-way ANOVA followed by LSD Post-Hoc test to determine the significance (*p < 0.05, **p < 0.005, ***p < 0.001).

Next, we investigated whether the observed inhibition of cell viability (Fig. 1A) upon RCE treatment was associated with induction of apoptosis. Annexin V staining showed an increase in apoptotic populations in a concentration-dependent manner starting at 300 µg/mL RCE indicating, that these cells underwent apoptosis (Fig. 5A and B). No necrotic cells were detected at all tested RCE concentrations (data not shown). To further confirm the induction of apoptosis in response to RCE in HT-29 cells, we looked for cleaved PARP by Western blotting. As shown in Fig. 5D, RCE induced a concentration-dependent increase in the level of cleaved PARP. Next, we assessed for the activation of the executioner caspase-3/7 using a caspase 3/7 activity assay as described in materials and methods section. As shown in Fig. 5C, concentrations of 300 and 450 µg/mL RCE led to a significant increase in caspase 3/7 activity by 3 and 5 folds, respectively. Western blot analysis showed that RCE caused a significant concentration-dependent decrease in the level of caspase 3. Strikingly, this decrease in the pro-form was not associated with an increase in the processed active form (Fig. 5D, middle panel). Based on this result, it appears that RCE-induced apoptosis is independent of caspase-3 activation. This prompted us to assess the activation of Caspase-7. As shown in Fig. 5D, an increase in the level of cleaved caspase-7 was obvious in RCE-treated HT-29 cells, suggesting that *Rhus coriaria* induces a caspase-7-dependent apoptosis in colon cancer cells.

### Inhibition of autophagy rescues *Rhus coriaria-* induced cell death of colon cancer cells

**Figure 6:**
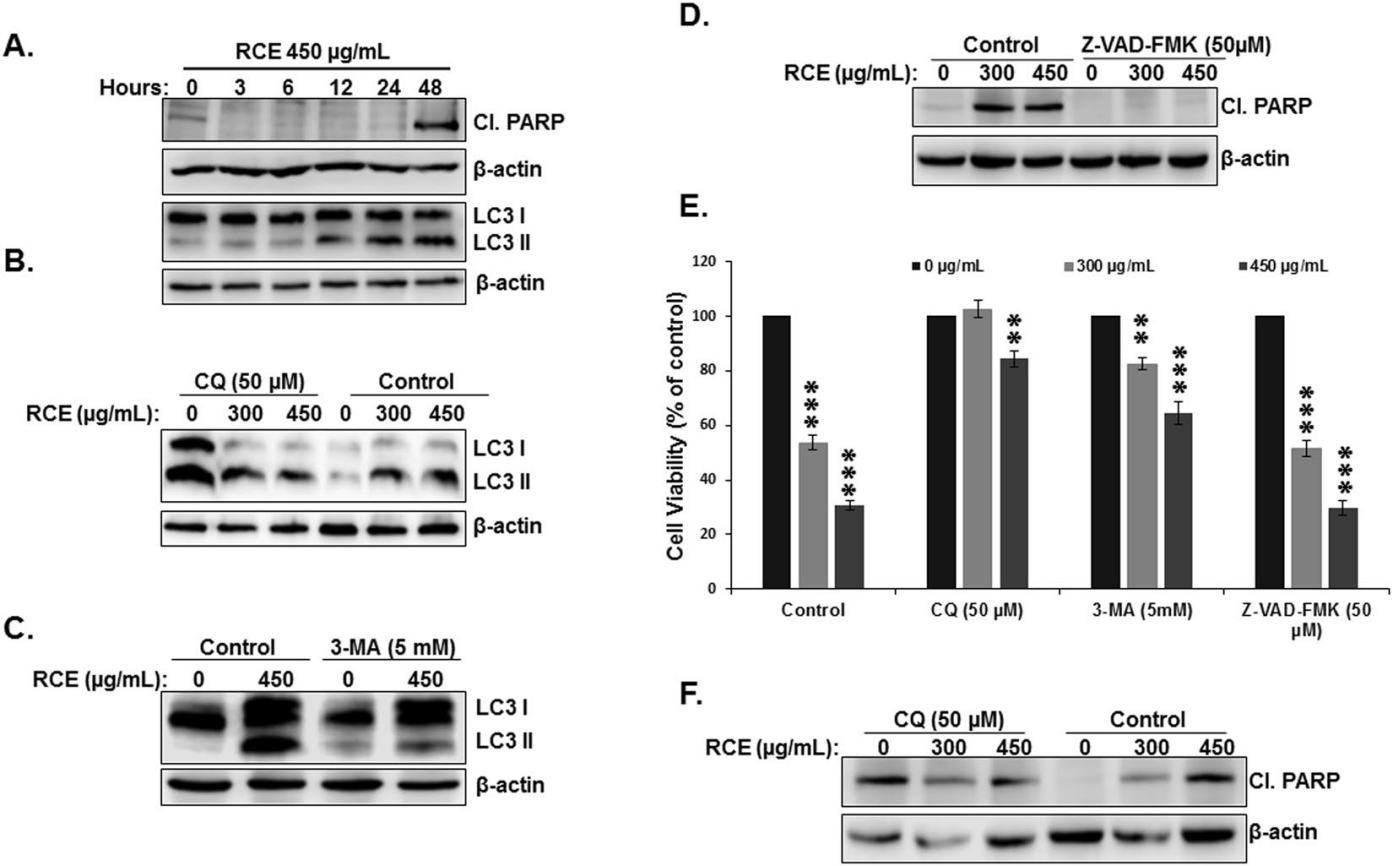
Autophagy precedes apoptosis and its inhibition, by autophagy inhibitors, rescues HT-29 from RCE-mediated cell death. (**A**) Time-course analysis, by Western blotting, of PARP cleavage and LC3-II accumulation in RCE-treated HT-29 cells. Cells were treated with 450 μg/mL RCE and proteins were extracted at the indicated time-points (3, 6, 12, 24 and 48 h) as described in Materials and Methods. (**B**,**C**) Analysis of LC3-II accumulation in HT-29 cells pre-treated with autophagy inhibitors. Cells were pretreated with or without CQ (50 µM) (**B**) or 3-MA (5 mM) (**C**) for 1 h and then RCE was added for 48 h. Proteins were extracted and LC3-II accumulation was determined by western blot. (**C**) Western blot quantification of cleaved PARP in cells pretreated with and without pancaspase inhibitors. (**E**) Inhibition of autophagy but not apoptosis reduces cell death induced by RCE. HT-29 cells were pretreated with CQ, 3-MA or the pan-caspase inhibitor (Z-VAD-FMK) as described above and then treated for 48 h with 300 or 450 µg/mL RCE. Cell viability was determined as described in Material and Methods. (**F**) Western blot of cleaved PARP in cells pretreated with and without autophagy inhibitor. Data are representative of six independent experiments. Statistical analysis for cell viability data on control or treated cells were performed using one-way ANOVA followed by LSD Post-Hoc test to determine the significance (**p < 0.005, ***p < 0.001).

Because RCE induced both apoptosis and autophagy, and because both events are known to induce cell death through type I and type II programmed cell death (PCD-I and PCD-II), respectively, we decided to investigate the contribution of those events (apoptosis and autophagy) to the cytotoxic activity of RCE. We first decided to determine the timing at which autophagy and apoptosis occurred. Toward this, we monitored the induction of autophagy and apoptosis over time by performing time-course analysis of both events. HT-29 cells were treated with 450 µg/mL RCE and autophagy was detected through the conversion of LC3-I into LC3-II while apoptosis was examined through PARP cleavage which is downstream of caspase activation. We found that while autophagy was evident after 12 hours post-treatment (Fig. 6A, lower panel), apoptosis (assessed by PARP cleavage) (Fig. 6A, upper panel), on the other hand, occurred 48 h post-treatment. These data clearly indicate that autophagy is an early event that precedes apoptosis induction in response to RCE.

*Rhus coriaria*, at concentration of 300 and 450 µg/mL, induces robust cell death (~60 and 70% inhibition of cell viability) after 48 h post-treatment (Fig. 1A) while apoptosis accounted for only ~16 and 25% as determined by Annexin V staining (Fig. 5A and B). This observation drove us to examine whether these two mechanisms of cell death are activated independently from each other or if they are linked events. To answer this question, we examined the effect, of CQ (inhibitor of autophagosomal degradation), 3-MA (inhibitor of autophagosome formation) and Z-VAD-FMK (pancaspase inhibitor) on cell viability. Blockade of autophagy was confirmed by evident decrease in the conversion of LC3-I to LC3-II by CQ (Fig. 6B) and 3-MA (Fig. 6C) whereas blockade of apoptosis was revealed by the absence of cleaved PARP (Fig. 6D). We found that cell viability was markedly and significantly improved when autophagy was inhibited compared to control cells treated with RCE only (Fig. 6E). Conversely, inhibition of apoptosis by Z-VAD-FMK had almost no effect on cell death when compared to control cells treated with RCE only (Fig. 6E) in HT-29 cells. This result is rather unexpected because at RCE concentrations of 300 and 450 µg/mL, apoptosis accounted for ~ 16 and 25% of cell death, respectively (Fig. 5A and B) in HT-29 cells. Similar result was obtained with Caco-2 cells (Figure S4B). It has been reported that blockade of apoptosis, by caspase inhibitor, lead to autophagic cell death^32, 33^. Therefore, we hypothesize that blockade of PCD-I in RCE-treated cells switches to the activation of PCD-II mechanism. It is also noteworthy to mention that, although CQ induced PARP cleavage in control cells, treatment with RCE, however, did not lead to a further increase of the level of cleaved PARP (Fig. 6F), demonstrating that the inhibition of autophagy caused the inhibition of RCE-induced apoptosis and thus suggesting that induction of apoptosis is autophagy-dependent. These results along with the time-course data, support the hypothesis that RCE-treated cells first underwent RCE-induced autophagy and later, probably due to longer exposure and/or excessive cellular damage, progressed to apoptosis.

### *Rhus coriaria* induces proteasome-dependent degradation of mTOR, Akt, p53 and caspase-3 in HT-29 colon cancer cells

**Figure 7:**
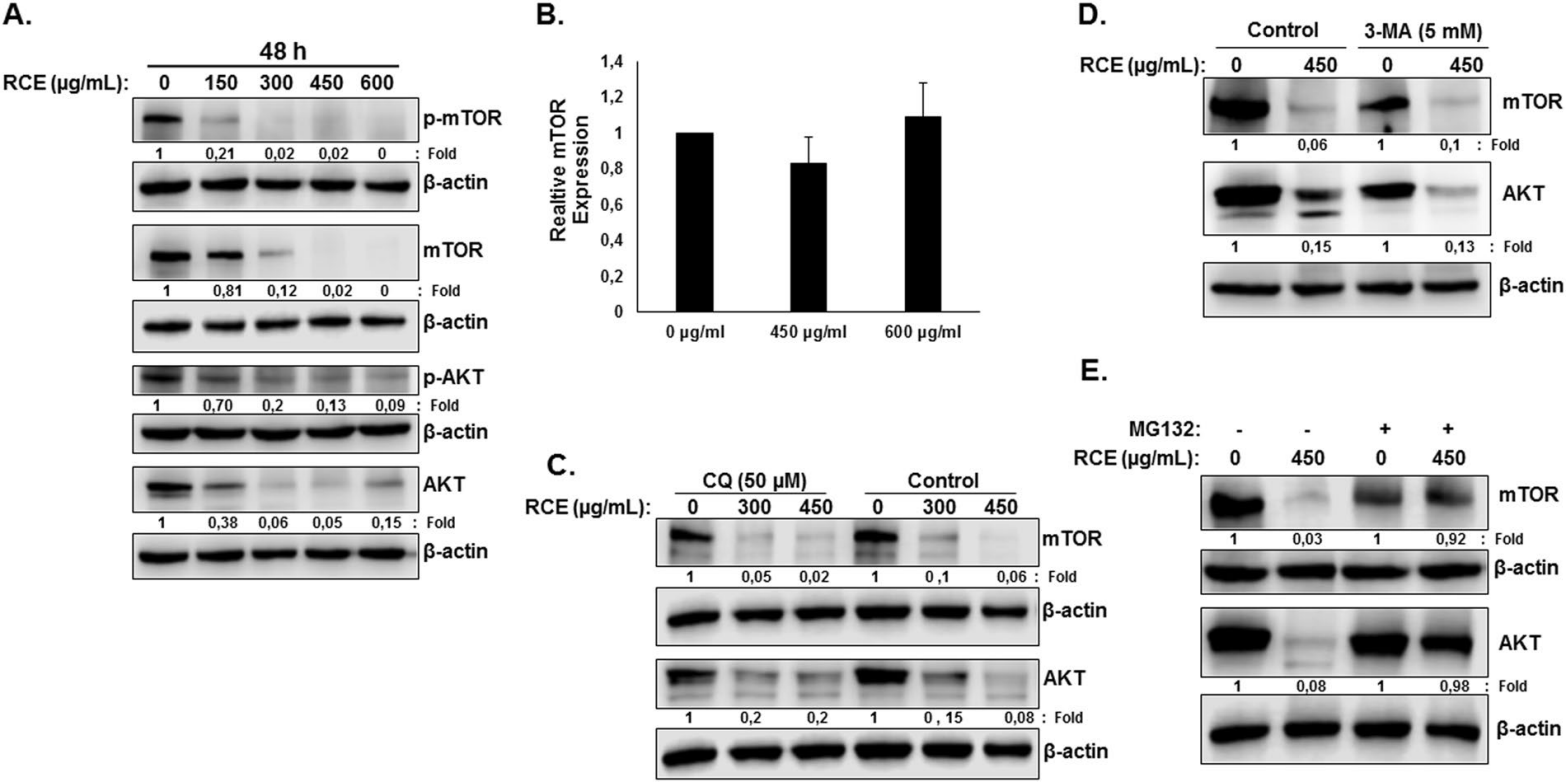
*Rhus coriaria* inhibits the AKT/mTOR pathway through targeting of mTOR and AKT proteins to proteasome-dependent degradation. (**A**) Concentration-dependent decrease of phospho-mTOR, total mTOR, phospho-AKT and total AKT protein in RCE-treated HT-29 cells. Cells were treated with or without increasing concentrations of RCE for 48 h, then whole-cell extracts were subjected to Western blot analysis for the phosphorylated and non-phosphorylated form of mTOR and AKT and for β-actin (loading control). (**B**) Downregulation of mTOR is transcription-independent. Total RNA from RCE-treated and untreated cells were used to amplify by qRT-PCR the mTOR transcripts using mTOR specific primers. GAPDH was used as internal normalization control. (**C,D**) RCE-mediated decrease in the protein level of mTOR and AKT is autophagy-independent. Cells were pretreated with or without CQ (50 µM) (**C**) or 3-MA (5 mM) (**D**) for 1 h and then RCE was added at the indicated concentration for 48 h. Proteins were extracted and mTOR and AKT protein level was determined by western blot. (**E**) RCE targets mTOR and AKT to proteasome degradation. HT-29 cells were pre-treated for 2 h with or without MG-132 (15 µM) prior to treatment with RCE (450 µg/mL). Whole cell lysate was resolved on 6% SDS-PAGE and analyzed for the two proteins.

Next, we examined the mechanism through which *Rhus coriaria* might exert its effects, particularly on autophagy and apoptosis. mTOR kinase, a downstream target of the PI3K/AKT signaling pathway and the major negative regulator of autophagy, was reported to regulate colorectal tumorigenesis^34^. We therefore decided to determine the effect of *Rhus coriaria* on the PI3K/AKT/mTOR pathway in HT-29 cells. Toward this, we first examined the level of phosphorylation of mTORC1 at Ser2448. Treatment with RCE led to a concentration-dependent dramatic decrease in the level of phosphorylation of mTORC1 in both HT-29 (Fig. 7A) and Caco-2 (Figure S5A) cells, suggesting an inhibition of mTOR activity in response to RCE. Surprisingly, a significant decrease in total mTOR protein level was also observed in both cell lines (Figs 7A and S5A) in response to RCE. Similarly, RCE also led to the decrease of phosphorylated and total AKT protein, the upstream regulator of mTOR pathway in HT-29 (Fig. 7A) and Caco-2 (Figure S5A) cells. qRT-PCR analysis of mTOR (Fig. 7B) and AKT (data not shown) mRNA transcript level showed no significant difference in mRNA levels between control and RCE-treated HT-29 cells. Also, blockade of late stage autophagy (autophagolysosome formation) by CQ (Fig. 7C) and early stage autophagy (autophagosome formation) by 3-MA (Fig. 7D) failed to restore mTOR and AKT protein levels, hence clearly demonstrating that decrease of these two proteins in response to RCE is not a result of autophagolysosomal degradation. To test if proteasomal activity is involved in RCE-mediated decrease of mTOR and AKT protein, HT-29 cells were first pre-treated with the proteasome inhibitor MG-132 and then treated with or without RCE. Results shown in Fig. 7E shows that MG-132 treatment was able to restore these two proteins to a level comparable to the control. Altogether, these findings suggest that mTOR and AKT reduction in RCE-treated cells is due to a proteasome-dependent degradation of these proteins which ultimately drives the cells toward autophagy.

**Figure 8:**
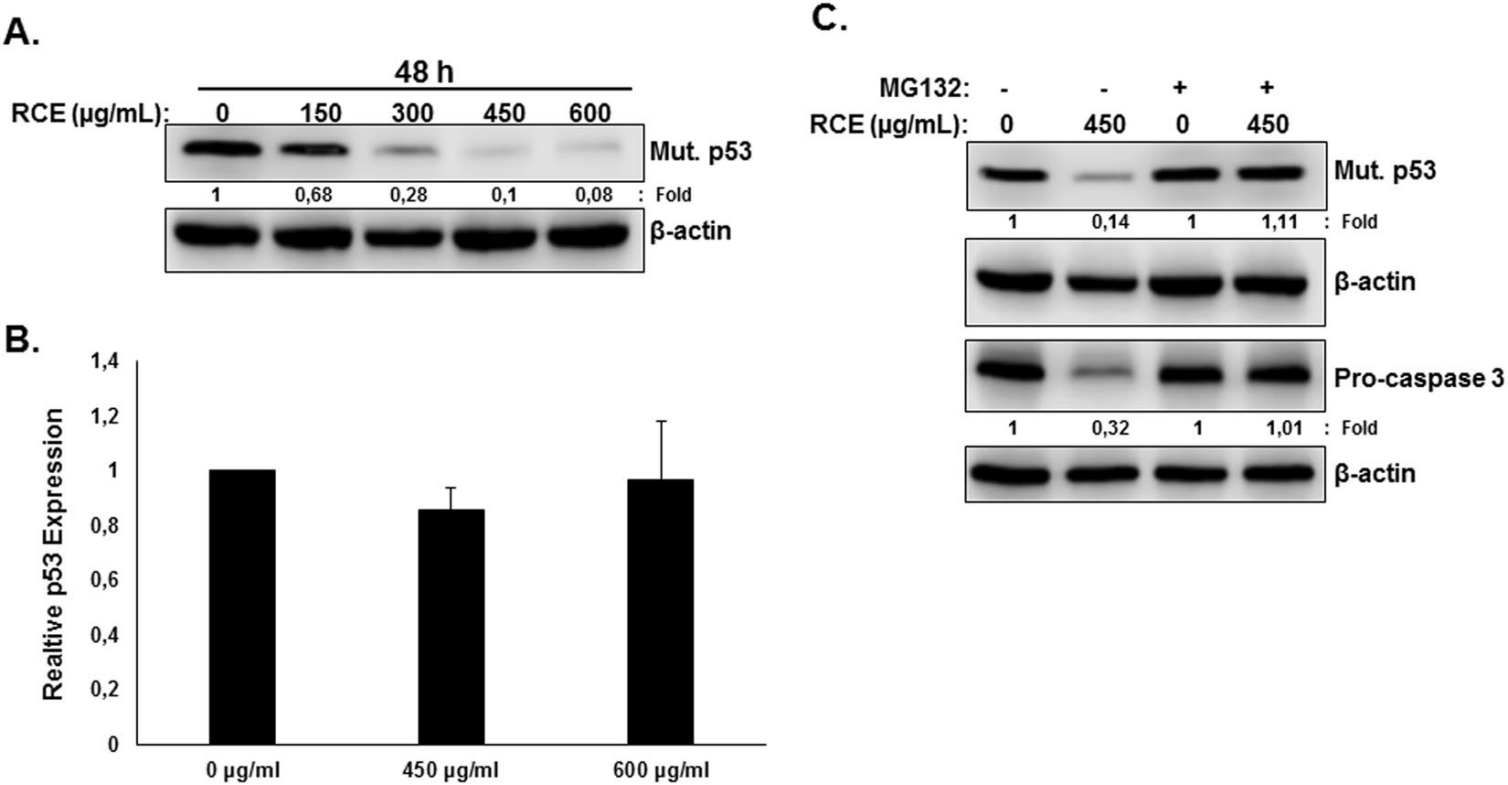
*Rhus coriaria* targets mutant p53 and procaspase-3 to proteasome degradation in HT-29 cells. (**A**) Downregulation of mutant p53 protein in RCE-treated cells. Cells were treated with or without increasing concentrations of RCE for 48 h, then whole-cell extracts were subjected to Western blot analysis for p53 protein. (**B**) qRT-PCR analysis of p53 transcript in RCE-treated cells. (**C**) RCE targets mutant p53 and procaspase-3 to proteasome degradation. HT-29 cells were pre-treated for 2 h with or without MG-132 (15 µM) prior to treatment with RCE (450 µg/mL). Whole cell lysate was resolved on 12.5% SDS-PAGE and analyzed for the p53 and procaspase-3 proteins.

Next, we examined protein level of mutant p53 in RCE-treated HT-29 cells. Results in Fig. 8A showed a concentration-dependent decrease in mutant p-53 protein. This decrease is not a result of reduced gene expression, as qRT-PCR analysis revealed no difference in mRNA level of p53 transcripts between control and treated cells (Fig. 8B). A concentration-dependent decrease in mutant p-53 protein was also observed in Caco-2 cells (Figure S5A). Interestingly, inhibition of the proteasome by MG-132 totally rescued mutant p53 from degradation (Fig. 8C), suggesting that mutant p53 is also targeted to proteasome degradation by *Rhus coriaria* in colon cancer cells.

We have demonstrated that RCE induced downregulation of procaspase 3 without concurrent increase in the active form (Fig. 5D). This prompted us to investigate whether procaspase 3 is also targeted to proteasome degradation. Indeed, we found that inhibition of proteasome by MG-132 restored procaspase 3 protein to a level comparable to control cells hence, indicating that procaspase 3 is also targeted for proteasomal degradation by RCE. Altogether, our results strongly argue in favor of activation of proteasomal degradation mediated by *Rhus coriaria* in colon cancer cells.

### *Rhus coriaria* stimulates the activation of proteasome-mediated proteolysis, associated with the degradation of mTORC1, leading to the activation of autophagy and subsequent apoptosis in colon cancer cells

**Figure 9:**
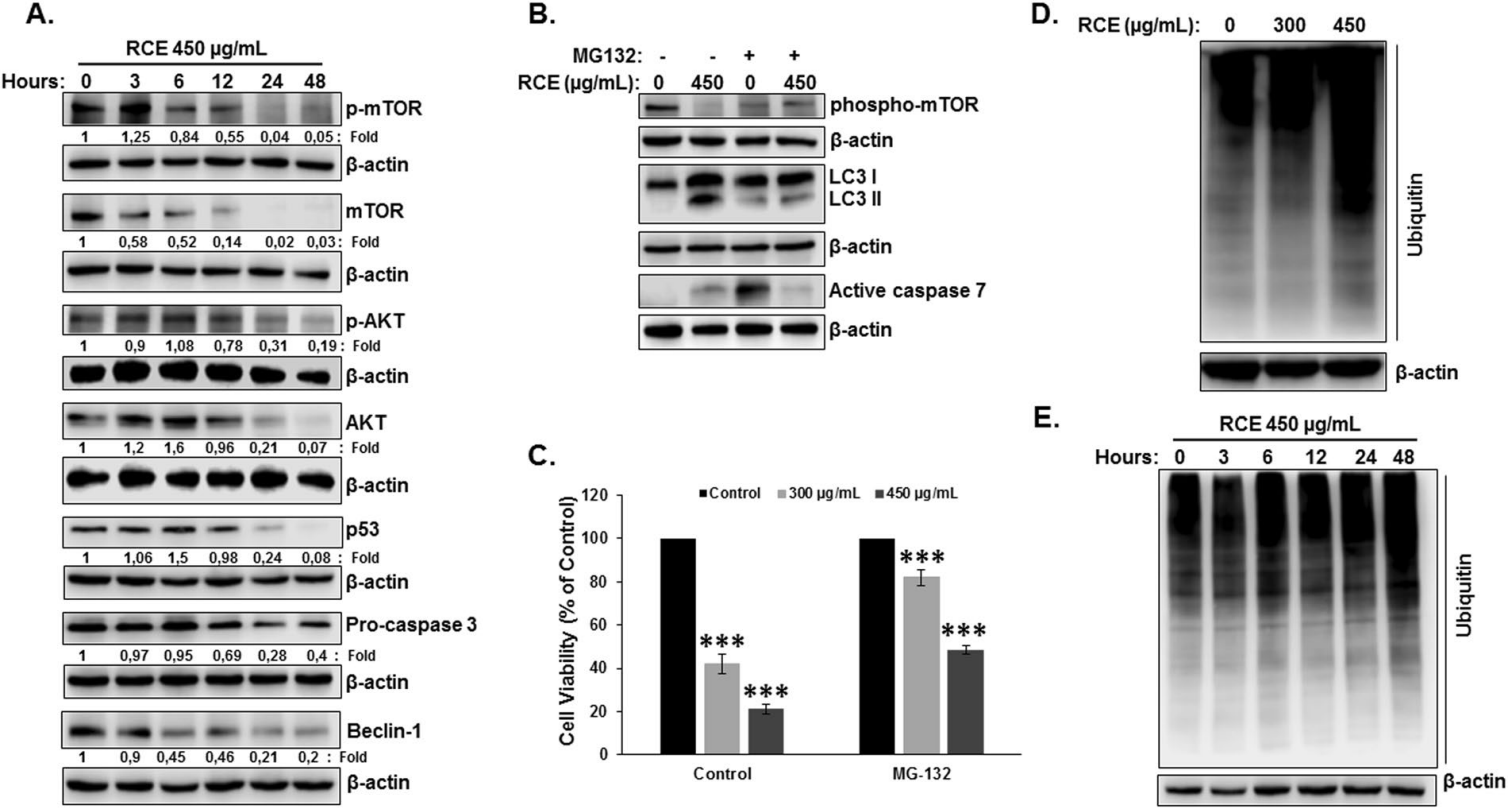
*Rhus coriaria* stimulates the activation of the ubiquitin proteasome system and its inhibition rescue blocks autophagy and apoptosis and rescues cell viability in HT-29 cells. (**A**) Inactivation of mTOR through proteasome degradation precedes autophagy. Time-course analysis, by Western blotting, of phospho-mTOR, total mTOR, phospho-AKT, total AKT, mutant p53 and procaspase-3 in RCE-treated HT-29 cells. Cells were treated with 450 μg/mL RCE and proteins were extracted at the indicated time-points (3, 6, 12, 24 and 48 h) as previously described. (**B**) Inhibition of the proteasome rescue phospho-mTOR and block autophagy and apoptosis induced by RCE. HT-29 cells were pre-treated for 2 h with or without MG-132 (15 µM) prior to treatment with RCE (450 µg/mL). Whole cell lysate was resolved on 6 and 15% SDS-PAGE for phospho-mTOR and LC3 and active caspase-7, respectively. (**C**) Inhibition of proteasome reduces cell death induced by RCE. HT-29 cells were pre-treated for 2 h with or without MG-132 (15 µM) prior to treatment with RCE (300 and 450 µg/mL) for 48 h. Cell viability was determined as described in Material and Methods. Data are representative of three independent experiments carried out in triplicate. Statistical analysis for cell viability data on control or treated cells were performed using one-way ANOVA followed by LSD Post-Hoc test to determine the significance (***p < 0.001). (**D**) RCE treatment increases the cellular level of ubiquitinated proteins in HT-29 cells. Cells were treated with or without RCE (300 and 450 µg/mL) for 48 h, then whole-cell extracts were subjected to Western blot analysis for ubiquitinated proteins. (**E**) Time-course analysis, by Western blotting, of protein ubiquitination in RCE-treated HT-29 cells. Cells were treated with 450 μg/mL RCE and proteins were extracted at the indicated time-points (3, 6, 12, 24 and 48 h) as previously described.

To determine the order of events at which autophagy and proteasome-dependent degradation of proteins occurs in HT-29 cells, a time course measurement of protein levels was carried out. We found that levels of mTOR protein and its phosphorylated form were first to decline. Indeed, total mTOR degradation occurred as early as 3 hours after RCE-treatment followed by a decrease in its active form detectable after 6 hours post-treatment (Fig. 9A). On the other hand, the level of AKT and its active form, p53 and pro-caspase 3 proteins started decreasing after 12 hours post-RCE treatment (Fig. 9A). Interestingly, we also showed that Beclin-1 downregulation, which occurred as early as 6 hours post-RCE treatment (Fig. 9A, lower panel), preceded autophagy which was triggered after 12 hours post-RCE treatment as determined by LC3-II accumulation (Fig. 6A). Altogether, our finding suggests that mTOR inactivation, through degradation, might serve as a trigger for downstream event (proteasomal degradation, autophagy and apoptosis) induced by *Rhus coriaria*.

To test this hypothesis, we first examined whether the rescued mTOR protein from proteasome degradation, by MG-132, (Fig. 7E, upper panel) could be phosphorylated, i.e. active. As shown in Fig. 9B (upper panel), inhibition of the proteasomal machinery restored phosphorylated mTOR to a level comparable to control cells. Having shown that, we next addressed whether the restoration of active mTOR has an impact on autophagy activation and subsequently on apoptosis induction. Results shown in Fig. 9B (upper panel) shows that indeed, restoration of phospho-mTOR was associated not only with a significant decrease in the conversion of LC3-I to LC3-II (Fig. 9B, middle panel), but also with reduced level of active caspase 7 (Fig. 9B, lower panel) indicative of reduced autophagy and apoptosis, respectively. To further confirm the blockade of autophagy and apoptosis, cell viability was measured in cell treated first with MG-132 and then with RCE. Interestingly, we found that cell viability was significantly improved after proteasomal inhibition (Fig. 9C). Altogether, our results strongly suggest that RCE-mediated activation of proteasomal degradation of mTOR and other proteins is the driving force that leads toward cell death through induction of autophagy and apoptosis in HT-29 cells.

Recent work showed that a decrease in mTOR activity increases overall protein ubiquitination and degradation by the ubiquitin proteasome system^35^. This prompted us to examine whether inactivation of mTOR activity by RCE enhances overall protein ubiquitination. HT-29 cells were treated with 300 and 450 μg/mL RCE and overall protein ubiquitination level was determined by Western blotting using ubiquitin antibody. We found that treatment with RCE resulted in marked increase in the total content of ubiquitinated protein (Fig. 9D). An increase in the level of ubiquitinated protein was also observed in Caco-2 cells (Figure S5B). Moreover, a time course experiment revealed that the increase in the level of protein ubiquitination could be detected as early as 3 hours which also coincides with a time at which a decrease of mTOR protein was observed (Fig. 9E).

### *Rhus coriaria* slows down tumor growth in HT-29 mouse xenograft

**Figure 10:**
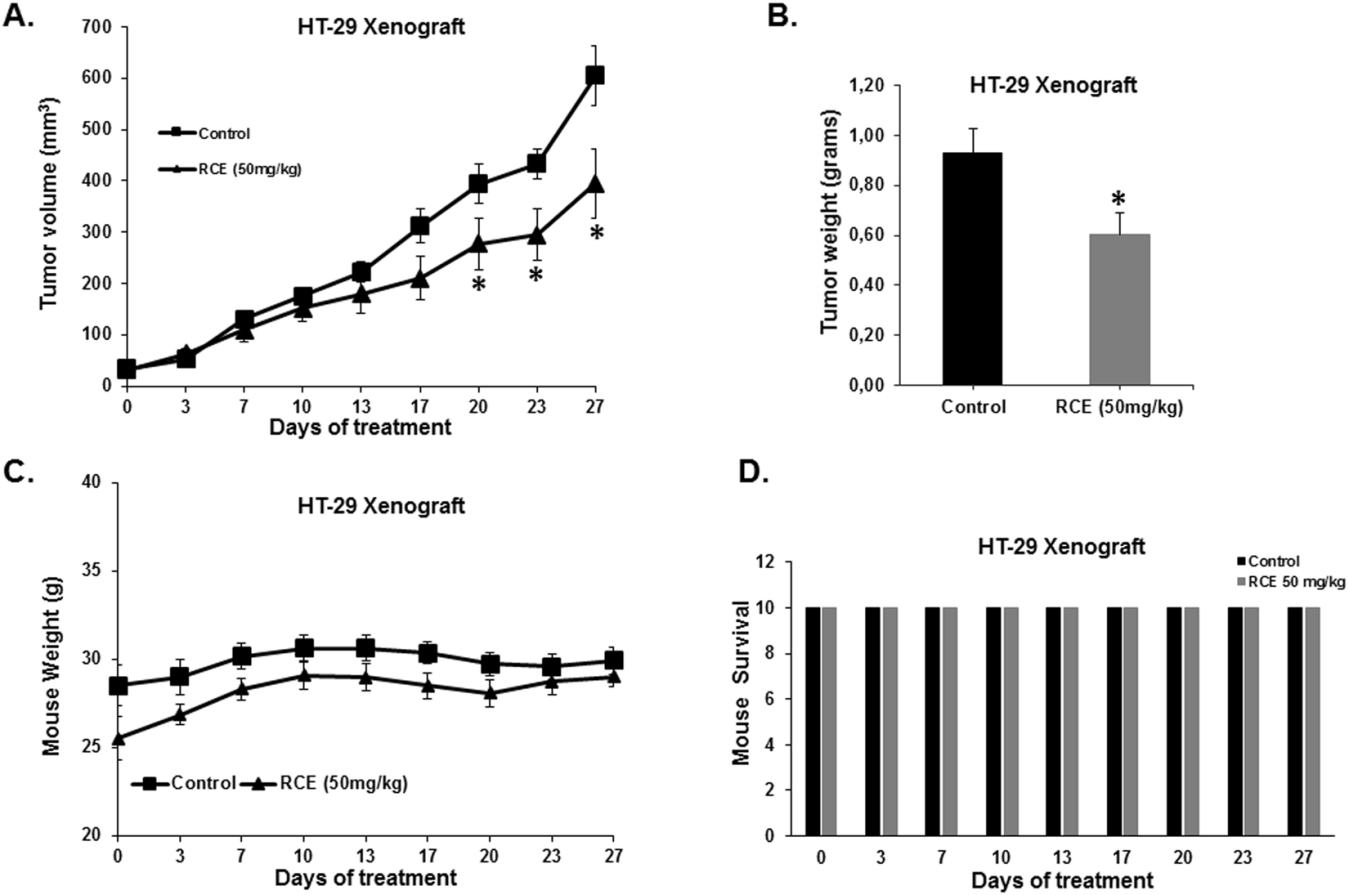
Effect of RCE on tumor growth in HT-29 tumor xenografts. **(A**) Tumor volume of HT-29 xenografts inoculated subcutaneously in nude mice and treated with RCE (50 mg/kg, intra-peritoneal injections) or control carrier solution alone, for a total of 27 days. (**B**) Tumor weight obtained from the same control and treated nude mice. (**C)** Body weight of these mice. (**D**) Toxicity screening of RCE-treatment on mouse survival. Toxicity was evaluated by comparing the number of life mouse in control and RCE-treated mice. Data points represent the mean ± S.E.M. of 10 mice per group. *Significantly different at p < 0.05.

To confirm the pharmacological relevance of our *in vitro* data, the anticancer activity of RCE was investigated *in vivo* in athymic mice inoculated with HT-29 colon cancer cells. The growth of the HT-29 human tumor xenografts was monitored every third or fourth day for 27 consecutive days after daily i.p. injection of 50 mg/kg of RCE. Treatment reduced the volume of the HT-29 xenografts by 35% (p < 0.05) (Fig. 10A) and tumor weight at the end of the experiment by 35% as well (p < 0.05) (Fig. 10B). There were no manifest undesirable effects of RCE treatment on body weight (Fig. 10C). In addition, there were no visible abnormalities at necropsy, or any other obvious signs of toxicity like behavioral changes. Toxicity was also evaluated by comparing the number of life mouse in control and RCE-treated. At the end of the experiment (Day 27), RCE showed no cytotoxicity as there was no difference in the number of surviving mouse in control and RCE treatment (Fig. 10D).

## Discussion

Increasing evidence indicates that herbal extracts and their compounds can inhibit cell growth and promote cancer cell death through different mechanisms including autophagy and apoptosis. In our recently published study, we showed that *Rhus coriaria* extract could promote autophagy^14^ and suppress angiogenesis, metastasis and tumor growth of highly invasive and metastatic breast cancer^15^. Importantly, *R*. *coriaria* was shown to be safe to consume by both humans and animals. In fact, rats fed with doses up to 1 g/kg of lyophilized extract showed no signs of toxicity or mortality^36^. In the present study, we examined the potential anticancer activity of *Rhus coriaria* on colorectal cancer. Our findings demonstrate that *R*. *coriaria* inhibits the viability and colony growth of colon cancer cells through inactivation of proteasome-dependent degradation of mTOR. Indeed, we found that *Rhus coriari*a treatment first, stimulates the intracellular protein ubiquitination and proteasome degradation of proteins including caspase-3, AKT and p53 in a concentration-dependent manner. Furthermore, we found that this early event serves as a trigger for the induction of non-canonical Beclin-1-independent autophagy and subsequent autophagy-dependent caspase-7-dependent apoptosis which ultimately leads to cellular death of colon cancer cells.

A well-defined upstream regulator of mTORC1 is the phosphoinositide 3-kinase-AKT-mammalian target of rapamycin (PI3K/AKT/mTOR) signaling pathway, although the activation of mTORC1 can also occur through an AKT-independent pathway^37^. Activated AKT directly phosphorylates and consequently inhibits TSC1/2 leading to activation of mTORC1. Activated mTOR coordinates the overall protein turnover and thus promoting cell growth and proliferation^38^. Added to that, mTOR pathway negatively regulates autophagy, and the inactivation of mTOR is looked at as a crucial step in autophagy activation. The PI3K/AKT/mTOR pathway is frequently hyperactivated in many cancers, including colorectal cancer^39^, and is important for aggressive tumor growth and cell survival. Recently, inhibition of this pathway through genetic/chemical inhibition of mTORC1 has emerged as potential target for colorectal cancer. Indeed stable knock down of mTOR inhibited tumorigenesis in mouse model^34^. Rapamycin, the first discovered natural inhibitor of mTOR, was shown to inhibit the proliferation of rapamycin sensitive colorectal cancer cell lines^34^. Recently, aspirin was also reported to exert a protective effects against development of colon cancer cells through inhibition of mTOR and induction of autophagy in colorectal cancer cells^40^. Here we showed that RCE targeted both AKT and mTOR to proteasome-dependent degradation, along with other proteins. Interestingly, we found that mTOR degradation occurred as early as 3 hours post-treatment and was concomitant with an increase in the level of protein ubiquitination while the depletion of AKT occurred only 12 hours post-RCE treatment, a timing at which autophagy was induced. This point out that suppression of mTOR occurs through an AKT-independent mechanism. These observations also strongly suggest that RCE-mediated effects in colon cancer cells may be initiated, at least partly, through degradation and hence inactivation of mTOR. Our data are in agreement with this claim, since the inhibition of the proteasome by MG-132 not only restored total mTOR level but also restored phosphorylated mTORC1, blocked autophagy and attenuated cell death in HT-29 cells induced by *Rhus coriaria*. To our knowledge, we are the first to report inhibition of mTOR pathway through targeting of total mTOR protein to proteasome degradation in colorectal cancer cells.

Increasing number of anticancer therapies has been shown to induce autophagy in different cancer cell types^41^. Here, we showed that *Rhus coriaria* induced autophagy in a concentration-dependent manner in colon cancer cells. This finding is supported by bodies of evidence including intracellular cytoplasmic vacuolation, modulation of autophagy-specific markers such as conversion of LC3-I to LC3-II and modulation of p62 (SQSTM1) accumulation. This agrees with our previous finding on breast cancer cells in which *Rhus coriaria* was also shown to elicit autophagy^14^. Surprisingly, and in contrast to breast cancer in which RCE induces beclin-1 dependent autophagy^14^, in the present study we found that RCE induced Beclin1-independent autophagy in colon cancer cells. In fact, RCE promoted proteasome degradation of Beclin-1 and inhibition of the proteasome restored Beclin-1 to a level comparable to non-treated control cells. Similar findings were made for the natural compound resveratrol which was shown to induce canonical autophagy in human colorectal cancer cells^42^ and non-canonical Beclin-1 independent autophagy in breast cancer cells^43^. It seems then that the type of autophagy canonical and non-canonical induced in response to anticancer drugs depends mainly on the cell type. Still, whether autophagy in response to anticancer therapies is pro-death or pro-survival remains subject to debate. There are increasing evidences that non-canonical Beclin1-independent autophagy is invariably associated with cell death referred as type II programed cell death (PCD II)^43, 44^, thus in agreement with our finding. Increasing number of studies showed that autophagosome formation occurs in the absence of key autophagy actors such as Beclin-1^45^. The non-canonical Beclin-1-independent autophagy has been reported in cell treated with compounds showing anticancer activities such as resveratrol^43^, carnosol^46^ and cobalt chloride^47^. Interestingly, Sun and collaborators showed that non-canonical Beclin-1-independent autophagy was induced in Beclin-1-depleted HeLa cells in response to cobalt chloride^47^. Similarly, we found, in the present study, that RCE induced both depletion of Beclin-1 through proteasome degradation and Beclin-1-independent autophagy in colon cancer cells. Based on these findings, we can deduce that non-canonical autophagy can be induced when the function of canonical autophagy proteins is compromised.

Apoptosis and autophagy are considered two different events; cross-talk between autophagy and apoptosis exists and the intricate interplay between these two mechanisms is a big challenge for cancer treatment. Autophagy seems to play a role in cancer cell survival and cell death. It contributes to cytoprotective events that help cancer cells to survive and to protect cells from apoptosis^48^. In other circumstances, autophagy can stimulate a pro-death signal pathway in cancer cells. Moreover, under some situations, apoptosis and autophagy can exert synergetic effects, whereas in other conditions autophagy can be triggered only when apoptosis is suppressed^48^. Hence, the question whether autophagy elicits or inhibits apoptosis may depend upon the cell type, nature and duration of stimulus^49^. Here we showed that although apoptosis is activated in response to RCE, it does not constitute the main mechanism of cell death and cell death occurs mainly through PCD-II probably as result of excessive autophagy. This claim is supported by several evidences. First, we showed that autophagy preceded apoptosis. Indeed, while activation of autophagy occurred as early as 12 hours and reduced cell viability was observed already after 24 h post-RCE treatment, apoptosis on the other hand was apparent after 48 hours, revealed by caspase-7 activation and PARP cleavage. Moreover, chemical inhibition of autophagy by CQ abrogated RCE-induced cell death while inhibition of apoptosis by the pan-caspase inhibitor had no effect on cell death. Our results here revealed that even though caspase-3 is markedly depleted in RCE-treated HT-29 cells, due to its proteasome-dependent degradation, the mechanism for apoptosis is still functional. Indeed, we showed that these cells were able to induce apoptosis through an alternate caspase-7 dependent pathway. Similarly induction of caspase-7-dependent autophagy was observed in caspase-3 deficient breast cancer cells when treated with tyrylpyrone derivative (SPD), a plant-derived pharmacologically active compound extracted from *Goniothalamus sp*.^50^. We believe that cell death through activation of apoptosis, although minimal, comes as secondary response due to increased intracellular stresses and therefore accumulation of cellular damage due to longer exposure of the cells to RCE.

The balance between the regulation of protein turnover and nutrient availability determines the overall status of cell growth. When nutrients are abundant, protein synthesis rates elevate, while protein degradation are kept to minimal. Whereas in energy-stressed cells, synthesis drops with rise in overall degradation^51^. One crucial factor in the coordination of overall protein turnover is mTOR, which promotes growth and suppresses autophagy^38^.

mTOR inhibition, due to cell starvation or direct experimental inhibition, is known to induce autophagy and stimulates protein breakdown^17^. Very recent studies have established that proteolysis through the ubiquitin proteasome system (UPS) is also regulated by mTORC1. Conversely, it is yet to be concluded whether it stimulates or suppresses the UPS activities, due to the contradictory nature of these studies, where the first study reported that inhibition of mTORC1 reduced proteolysis through suppressing the expression of proteasome^52^, whilst the second study by Zhao and collaborators reported the opposite, sighting that the inhibition of mTOR stimulates and enhances both proteolysis by UPS and autophagy^35^. Our results coincide with the later. Indeed, we found that the earliest effect induced by *Rhus coriaria*, and noticeable as early as 3 hours post-treatment, was an increase in the overall level of protein ubiquitination and the degradation of total mTOR protein. The mechanism through which inhibition of mTOR stimulates proteasome degradation deserves further investigations.

Until recently, ubiquitin-proteasome and autophagy degradation pathways were considered as independent events. However, recent studies pointed out that ubiquitination can target several proteins for degradation through both mechanisms^28^. In our case, it is tempting to speculate that autophagy may be activated as secondary pro-survival event that serves as back-up mechanism to help removing aggregated or misfolded proteins when the function of the proteasome system is overwhelmed due to excessive accumulation of damages as result of longer exposure to *Rhus coriaria*. In fact, we demonstrated that autophagy is blocked when proteasome function is inhibited by MG-132.

The tumor suppressor protein, p53, mutated in about 50% of human cancers^53^ was reported to gain new oncogenic functions such as chemoresistance to commonly used anticancer drugs, enhanced cell growth, metabolism and invasion^54, 55^ and, promoting the removal of mutant p53 might have therapeutic relevance in cancer cells. Studies have shown that depletion of mutant p53 by RNA interference in colon cancer cells reduces cell proliferation, *in vitro* and *in vivo* tumorigenicity and sensitizes cancer to anticancer drugs^56^. Hence, mutant p53 should be considered as a potential cancer-specific target for pharmacologic interventions in human cancers^57, 58^. Interestingly, mutant p53 was shown to be very stable, probably due to evasion from proteasome-dependent degradation, allowing its accumulation in cancer cells in response to stresses and this accumulation seems to play a key role for its oncogenic gain of functions that contribute to cancer development and progression^59^. Also, *in vivo* studies showed that cellular stresses such oxidative stress and DNA damage promote stabilization of mutant p53 required for its oncogenic function^60, 61^. A strategy, among others, that is currently being explored to inhibit mutant p53 function involves its targeting to degradation through the proteasome and autophagy pathways^55^. Here, we showed that *Rhus coriaria* promoted the proteasome-dependent degradation of p53 in colon cancer cells. Indeed, chemical inhibition of the proteasome activity by MG-132 not only rescued p53 from degradation but also significantly reduced *Rhus coriaria*-induced cell death. However, inhibition of autophagy had no effect on p53 protein level. It is noteworthy to mention that we have recently showed that *Rhus coriaria* also induced a dramatic decrease in the mutant p53 protein level in breast cancer cells^14^ raising the possibility that downregulation of mutant p53 protein level in breast cancer might have occurred through similar mechanism. Recent work by Yan and collaborators showed that arsenic trioxide targets mutant p53 to proteasome-dependent degradation in cancer cells through a mechanism involving in part induction of Pirh2 E3 ligase. Although still unknown, the exact molecular mechanism through which *Rhus coriaria* targets mutant p53 to proteasome-dependent degradation deserves further exploration.

**Figure 11:**
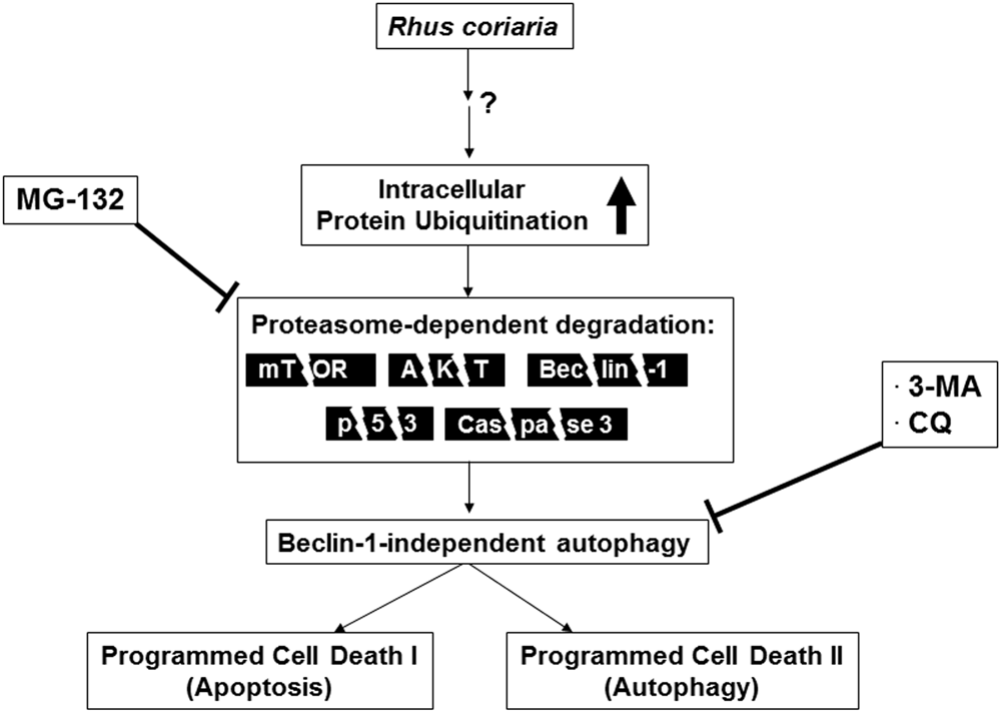
Hypothetic model for the anti-colon cancer effect of *Rhus coriaria*.

In summary, our data are consistent with the hypothetic model shown in Fig. 11 which shows for the first time the effect of *Rhus coriaria* extract on mutant p53 colon cancer cells. *Rhus coriaria*, through a yet to be elucidated mechanism, stimulates overall intracellular protein ubiquitination associated with proteasome degradation of component of negative regulator of autophagy pathway, namely the AKT and mTOR, Beclin-1, mutant p53 and caspase-3 which ultimately leads to induction of non- canonical Beclin-1-independent autophagy and subsequent caspase-7 dependent apoptosis. Our findings also provide a supplementary argument supporting the model that inhibition of mTOR stimulates proteasomal protein degradation and establish a link between the ubiquitin proteasome system and autophagy.

## Materials and Methods

### Cell culture, chemicals and antibodies

Human colon cancer cells HT-29 and Caco-2 were maintained in DMEM (Hyclone, Cramlington, UK) supplemented with 10% fetal bovine serum (FBS) (Hyclone, Cramlington, UK), 100 U/ml penicillin/streptomycin (Hyclone, Cramlington, UK). MG-132 was obtained from Cell Signaling, 3-Methyl adenine (3-MA) from Millipore (Hayward, CA, USA) and Chloroquine (CQ) from Sigma-Aldrich (Saint-Quentin Fallavier, France). Antibodies against LC3, AKT, Beclin-1, Ubiquitin, mTOR, phospho-mTOR (Ser 2448), caspase-3, caspase-7, and p53 were obtained from Cell Signaling (USA); those against cleaved caspase 3, and phospho-AKT (Ser 473) were obtained from Millipore (Millipore, Hayward, CA, USA), and those against p62/SQSTMI and cleaved PARP were obtained from Abcam (Abcam, Cambridge, UK). Antibody against PARP (full-length and cleaved) was purchased from BD Pharmingen. Antibody against β-actin was obtained from Santa Cruz Biotechnology, Inc (USA). Beclin-1 and control siRNA were purchased from Cell Signaling.

### Preparation of *Rhus coriaria* Ethanolic Extract (RCE)

Fruits of *Rhus coriaria* L. were collected from a private farm located at 33°16′35.59″N and 35° 19′02.89″E. The farm is located in Ma’rakeh, Tyre, Lebanon and the approval of the owner was obtained before collecting the fruit. This plant is readily and commercially available in the market. RCE was prepared as previously described^14^. Briefly, 10.0 g of the dried fruit were ground to a fine powder using a porcelain mortar and pestle. The powder was then suspended in 100 mL of 70% absolute ethanol and the mixture was kept in the dark for 72 hours at 4 °C in a refrigerator without stirring. After that, the mixture was filtered through a glass sintered funnel and the filtrate was evaporated to dryness using a rota-vapor at room temperature. The red residue was kept under vacuum for 2–3 hours and its mass was recorded. The residue was stored at −20 °C until further use.

### Measurement of cellular viability

Cells were seeded in triplicate in 96-well plates at a density of 7,000 cells/well. 24 hours later, cells were treated with or without various concentrations of RCE for different durations. Cell viability was measured with the Cell cytotoxicity assay kit (Abcam) according to the manufacturer’s instructioms. The results are representative of an average of at least 4 independent experiments. Data were presented as proportional viability (%) by comparing the treated group with the untreated cells, the viability of which is assumed to be 100%.

Cell viability was also measured with the Muse™ Cell Analyzer (Millipore, Hayward, CA, USA) using the Muse Count and Viability Kit (Millipore, Hayward, CA, USA) which differentially stains viable and dead cells based on their permeability to two DNA binding dyes. Briefly, cells were plated onto 12-well plates (50 × 10^4^ cells/ well). The day of treatment cells were counted to estimate the approximate number of cells per well. Following RCE treatment at indicated times, viable cells were counted using Muse™ Cell Analyzer.

### Colony formation assay

HT-29 cells were seeded in 6-well plate at a density of 2000 cells/well and allowed to grow for 7 days to form colonies before RCE is added. The growth media was replenished every 3 days. After 1 week, various concentrations of RCE were added in freshly added medium and the colonies were allowed to grow for 5 additional days. Colonies were photomicrographed at day 0 (colonies at day 7), three days (colonies at day 10) and 5 days (colonies at day 12) using Evos light microscope. Then, colonies were washed 3 times with PBS, fixed for 15 min with 4% formalin and stained with 0.01% crystal violet for 30 min. Colonies in each well were counted and their surface area was determined using the imageJ software. The experiment was carried in triplicate and repeated three times.

### Quantification of apoptosis by Annexin V labeling

Apoptosis was examined using the Annexin V & Dead Cell kit (Millipore, Hayward, CA, USA) according to the manufacturer’s instructions. Briefly, HT-29 cells were treated with or without RCE for 48 hours. Detached and adherent cells were collected and incubated with Annexin V and 7-AAD, a dead cell marker, for 20 min at room temperature in the dark. The events for live, early and late apoptotic cells were counted with the Muse™ Cell Analyzer (Millipore, Hayward, CA, USA).

### Quantification of caspase 3/7 activity: Caspase 3/7 activity

HT-29 cells were seeded at a density of 5,000 cells/well into 96-well plate in triplicate and treated with or without RCE for 48 h. Caspase-3/7 activity was measured using a luminescent caspase-Glo 3/7 assay kit (Promega Corporation, Madison, USA) following the manufacturer’s instructions. Briefly, caspase reagents were added to triplicate 96 wells. The plate was mixed on an orbital shaker and incubated for 2.5 h at room temperature in the dark. Luminescent signal was measured using the GloMax Multi-detectionSystem (Promega).

### Immunofluorescence staining

HT-29 cells (2 × 10^4^) were grown on 8 well labtek chamber slide (Becton Dickinson) for 24 h, then treated with or without RCE for 24 h. Cells were then fixed in 10% formalin solution (4% paraformaldehyde) (Sigma-Aldrich; Saint-Quentin Fallavier, France) for 5 min at room temperature followed by permeabilization in PBS containing 0.1% Triton X-100 for 5 min at room temperature (RT). Cells were then washed three times with PBS, blocked with 5% non-fat dry milk in PBS for 30 min at RT and then incubated with the primary antibody diluted in 1% non-fat dry milk/PBS for 2 h at 37 °C. Following incubation, cells were washed three times with PBS and incubated for 45 min at RT in the presence of fluorescein-conjugated secondary antibody diluted at 1:200 in 1% nonfat dry milk/PBS. After washing with PBS, cells were mounted in Fluoroschield with DAPI (Sigma-Aldrich) and examined under Olympus fluorescence microscope CKX53 (Olympus).

### Whole Cell extract and Western Blotting analysis

HT-29 Cells (6 × 10^6^) were seeded in 150 mm culture dishes and cultured for 24 hours before treatment. After incubation with RCE for the indicated time, cells were washed twice with ice-cold PBS, scraped, pelleted and lysed in RIPA buffer (Pierce) supplemented with protease inhibitor cocktail (Roche) and phosphatase inhibitor (Roche). After incubation for 30 min on ice, cell lysates were centrifuged at 14,000 rpm for 20 min at 4 °C. Protein concentration of lysates was determined by BCA protein assay kit (Thermo Scientific). Aliquots of 25 µg of total cell lysate were resolved onto 6–15% SDS-PAGE along with PageRuler Plus Prestained Protein Ladder (Thermo Scientific). Proteins were transferred to nitrocellulose membranes (Thermo Scientific) and blocked for 1 hour at room temperature with 5% non-fat dry milk in TBST (TBS and 0.05% Tween 20). Incubation with specific primary antibodies was performed in blocking buffer overnight at 4 °C. Horseradish peroxidase-conjugated anti-IgG was used as secondary antibody. Immunoreactive bands were detected by ECL chemiluminescent substrate (Thermo Scientific) and chemiluminescence was detected using the LiCOR C-DiGit blot scanner. Where needed, membranes were stripped in Restore western blot stripping buffer (Thermo Scientific) per the manufacturer’s instructions. Protein quantification was carried out using the ImageJ software.

### Knockdown of Beclin-1

HT-29 cells (250,000) were seeded in 6-well cell culture plate in serum-containing growth media and allowed to grow to 50% confluency. Then, cells were transfected with siRNA I (100 nM) using lipofectamine 2000 transfection reagent as described by the manufacturer (Invitrogen, Life technologies) for 48 h at 37 °C in 5% CO2 before treatment for 48 h with and without 300 and 450 µg/mL RCE in fresh complete media.

### RNA extraction and qRT-PCR

Total RNA from vehicle- or RCE-treated HT-29 cells were prepared using Trizol reagent as described by the manufacturer (Life Technologies, Inc.). The expression of specific genes was determined by qRT-PCR using the GoTaq 1-Step RT-qPCR system (Promega, Madison, USA) as per the manufacturer’s instructions. Amplification was carried out on the Stratagene Mx3000 P (Agilent technology). Briefly, the amplification reaction consisted of 100ng of total RNA and 0.2 µM primers in a final volume of 25 µl reaction. GAPDH was used as an endogenous reference for normalization. Expression levels were calculated by the comparative cycle threshold method, and normalization to the control was performed. A minimum of three technical replicates was used for each sample. Primer sequences are as follow: GAPDH(Forward): 5′-cacccactcctccacctttg-3′; GAPDH(Reverse): 5′-ccaccaccctgttgctgtag-3′; mTOR (Forward): 5′-ctgggactcaaatgtgtgcagttc-3′; mTOR(Reverse): 5′-gaacaatagggtgaatgatccggg-3′; Beclin-1(Forward): 5′-acagtggacagtttggcaca-3′; Beclin-1 (Reverse): 5′-cggcagctccttagatttgt-3; p53 (Forward): 5′-gttccgagagctgaatgagg-3′; p53 (Reverse): 5′-ttatggcgggaggtagactg-3′.

### Tumor growth assay

The animal experiments were performed in accordance with the protocol approved by the animal ethics committee at the UAE University (permit N° A22-14). Six-week-old athymic NMRI female nude mice (nu/nu, Charles River, Germany) were housed in filtered-air laminar flow cabinets and handled under aseptic conditions. Procedures involving animals and their care were conducted in conformity with Institutional guidelines that are in compliance with College of Medicine & Health Sciences, national and international laws and policies (EEC Council Directive 86/609, OJ L 358, 1, December 12, 1987; and NIH Guide for Care and Use of Laboratory Animals, NIH Publication No. 85-23, 1985). HT-29 cells (1 × 10^6^ cells in 200 µl PBS) were injected subcutaneously into the lateral flank of the nude mice. Two week after inoculation, when tumors had reached the volume of approximately 30 mm^3^, animals (ten in each group) were treated for 27 days with RCE (50 mg/kg/day, ip, five days per week) or carrier solution (control) in order to determine the effect of RCE on tumor growth. Tumor dimensions and animal weights were measured every 3 days. Tumor volume (V) was calculated using the formula: V = 0.4 × a × b^2^, with “a” being the length and “b” the width of the tumor. After sacrifice, the tumors were excised and weighed.

### Statistical analysis

Statistical analysis were done using SPSS version 21. Data were reported as group mean ± SEM. The data were analyzed via one-way ANOVA followed by LSD’s Post-Hoc multiple comparison test. Significance for all statistical comparisons was set at p < 0.05.

## Electronic supplementary material

Supplementary Information
