# Supplementary material for: Rhus coriaria increases protein ubiquitination, proteasomal degradation and triggers non-canonical Beclin-1-independent autophagy and apoptotic cell death in colon cancer cells
Source: Sci Rep. 2017 Sep 14;7:11633. doi: 10.1038/s41598-017-11202-3 (PMC5599689; doi:10.1038/s41598-017-11202-3)
Supplement: Supplementary file 1 — Supplementary Information [file 41598_2017_11202_MOESM1_ESM.pdf]

Supplementary Information

Rhus coriaria increases protein ubquitination, proteasomal degradation and triggers non-canonical Beclin-1-independent autophagy and apoptotic cell death in colon cancer cells

Khawlah Athamneh[q1], Hussain El Hasasna[q1], Halima Al Samri[1], Samir Attoub[2], Kholoud Arafat[2], Nehla Benhalilou[1], Asma Al Rashedi[1], Yusra Al Dhaheri[1], Synan AbuQamar[1], Ali Eid[3] and Rabah Iratni[1*]

[1]*Department of Biology, College of Science, United Arab Emirates University, Al-Ain, P.O. Box 15551, United Arab Emirates.*

[2]*Department of Pharmacology & Therapeutics, College of Medicine & Health Sciences, United Arab Emirates University, Al-Ain, P.O. Box: 17666, United Arab Emirates*

[3]*Department of Pharmacology and Toxicology, Faculty of Medicine, American University of Beirut, Beirut, PO Box 11-0236, Lebanon*

[q] *Contributed equally* to this work

*****Corresponding author:

R. Iratni, Department of Biology, United Arab Emirates University, P.O. Box: 15551, Al Ain, United Arab Emirates. Phone: +971 3 713 6526/6731, Fax: +971 3 7134927, Email: R_iratni@uaeu.ac.ae

Supplementary Methods

Detection of autophagic vacuoles: HT-29 cells (2 X 10^4) were grown in 8 chambers slides (Millipore) followed by treatment with or without RCE for 24 h. Following treatment cells were washed and stained for autophagic vacuoles using the autophagy detection kit (Abcam, Cambridge, UK) according to the manufacturer's instructions. Fluorescent autophagic vacuoles were examined under Olympus fluorescence microscope CKX 53 (Olympus).

Colony formation assay in soft agar: Assays were performed in six-well plates as previously described [38]. The lower (base) layer consisted of 1 ml 2.4% Noble agar. The base layer was overlaid with a second layer consisted of 2.9 ml growth medium, 0.3% Noble agar, and 3 × 10^4 HT29 cells. Growth medium was then added and plates incubated at 37° C. Cells were allowed to grow to form colonies for 10 days before RCE was added. At day 13, colonies were treated with or without RCE (300 and 450 µg/mL) for 5 days. Following treatment, plates were washed twice with PBS and then colonies were fixed with 10% ice-cold methanol for 10 min and washed once with PBS. Colonies were allowed to stain for 1 h in solution containing 2% Giemsa. Colonies in each well were counted using the imageJ software. The experiment was carried in duplicate.

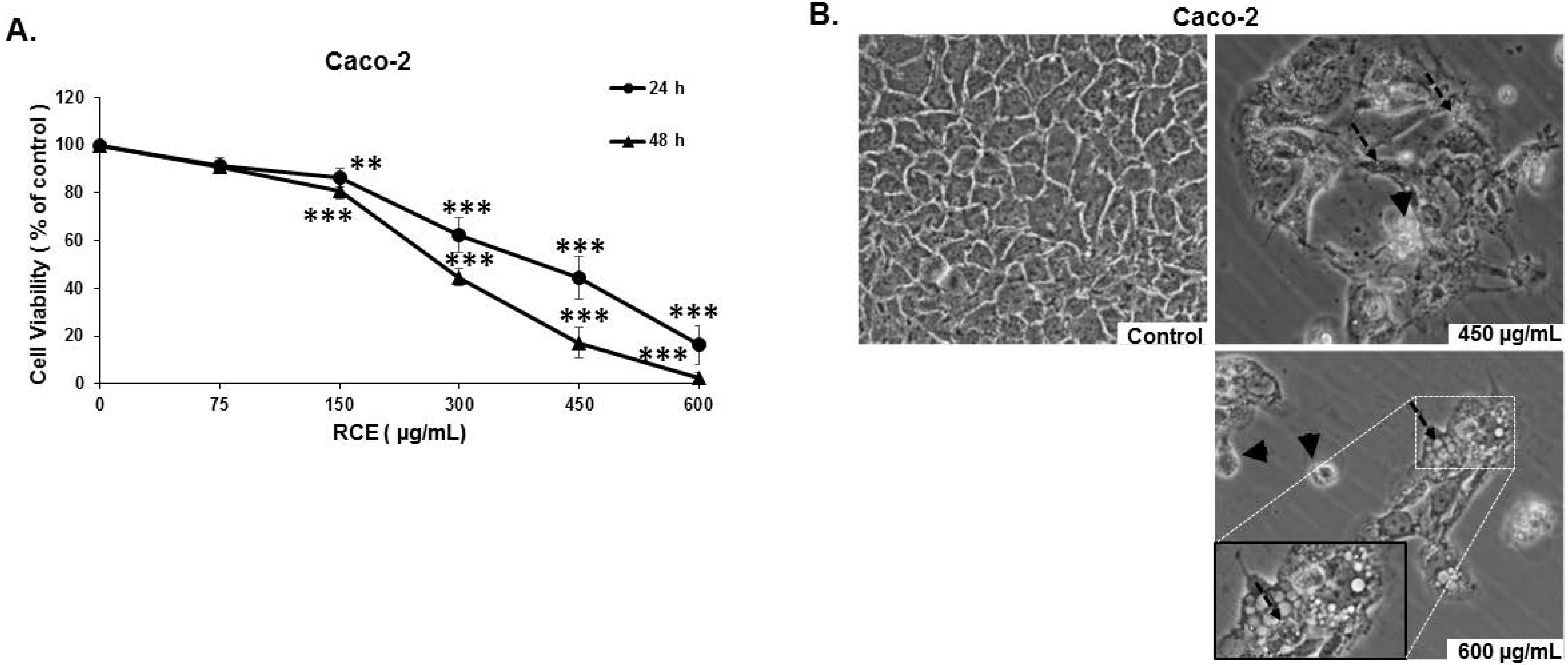

Figure S1. Inhibition of cellular viability of Caco-2 by Rhus coriaria. (**A**) Caco-2 colon cancer cells were treated with and without the indicated concentrations of RCE for 24 and 48. Viability was measured as described in Materials and Methods. Data represent the mean of three independent experiments carried out in triplicate. Statistical analysis for cell viability data on control or treated cells were performed using one-way ANOVA followed by LSD Post-Hoc test to determine the significance (***$p < 0.005$, ***$p < 0.001$*). (**B**) Morphological changes in RCE-treated Caco-2 cells. Morphological changes observed in the treated HT-29 cells after 48 h of treatment with the indicated concentration of RCE. Cells were observed under EVOS XL Core Cell Imaging System (Life Technologies) at 400X. Arrowheads shows intracellular vacuolation and dashed arrows shows dying cells. Statistical analysis for cell viability data on control or treated cells were performed using one-way ANOVA followed by LSD Post-Hoc test to determine the significance (***$p < 0.005$, ***$p < 0.001$*).

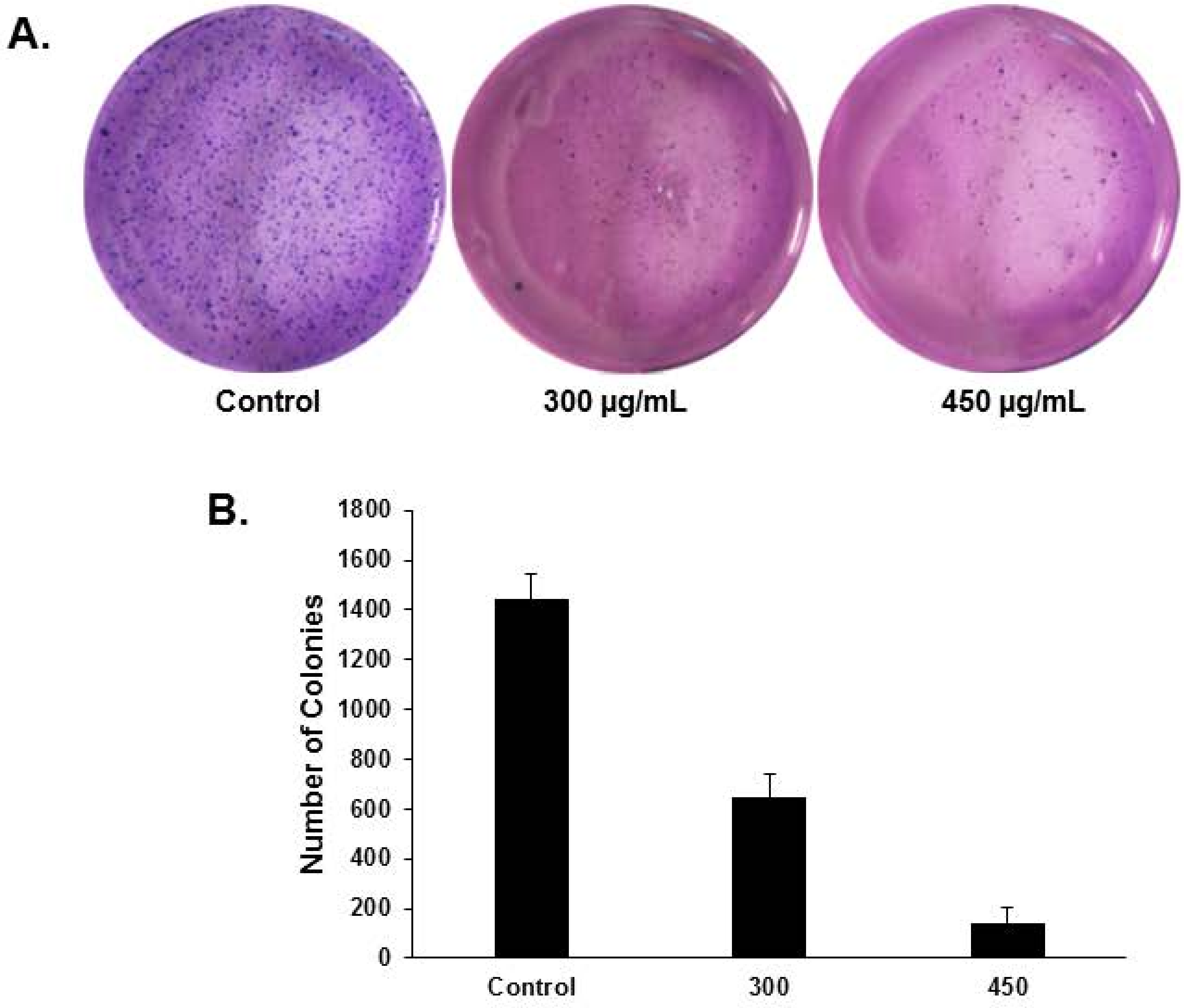

Figure S2. Rhus coriaria inhibits HT-29 colony growth in soft agar. HT-29 colonies were first allowed to form in normal media for 13 days as described in Material and Methods. Formed colonies (**A**) were then treated with or without RCE at the indicated concentrations and allowed to grow for 5 more days before staining. Inhibition of colony growth was assessed by measuring the size of the colonies obtained in control and RCE-treated plate using the imageJ software (**B**).

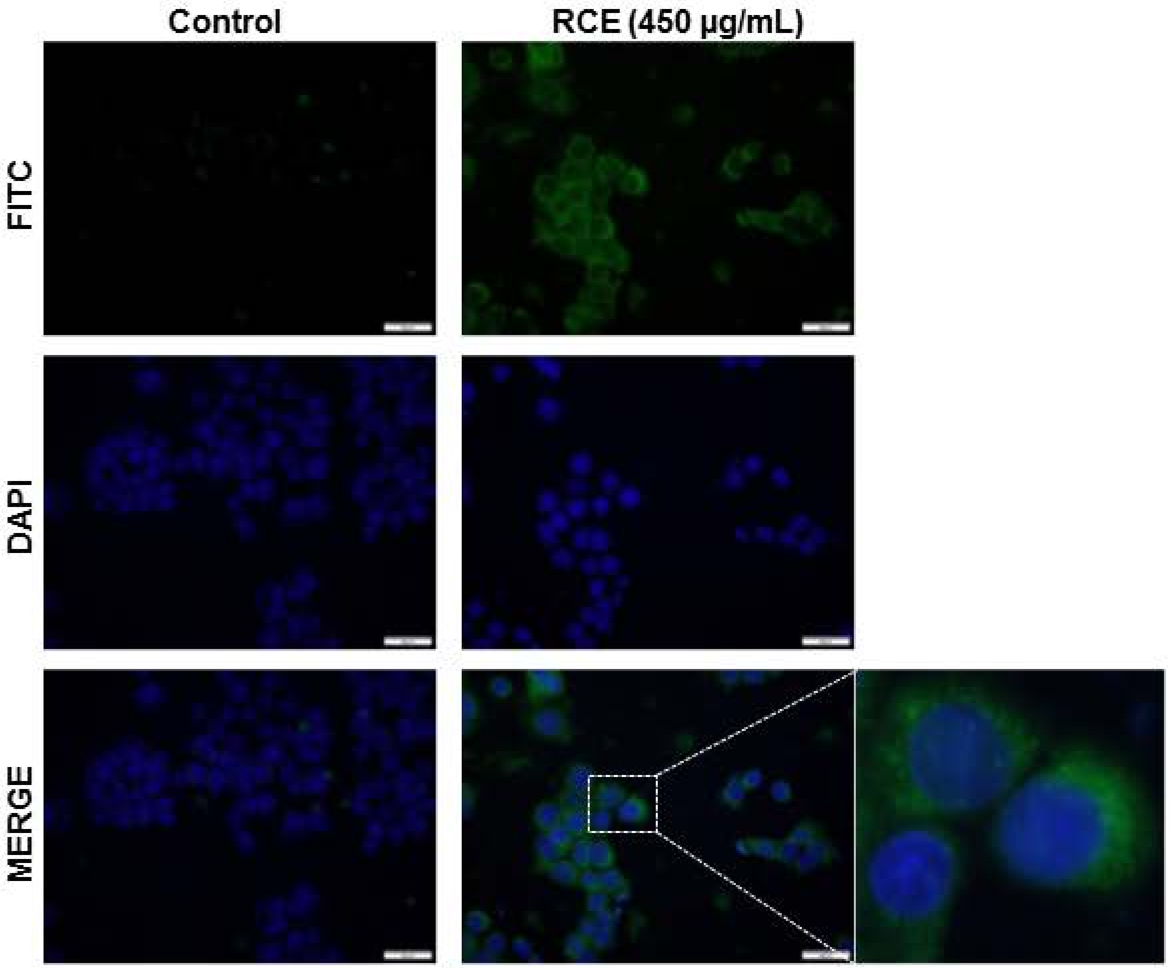

Figure S3. Rhus coriaria induces the formation of autophagic vacuoles in HT-29 cells. HT-29 cells cells were seeded at a density of 2×10⁴ cells/ chamber in 8 chambers slides (Millipore) followed by treatment with or without 450 µg/mL RCE. Following treatment cells were washed and stained for autophagic vacuoles as described in supplementary methods. Fluorescent autophagic vacuoles were examined under Olympus CKX53 fluorescence microscope (Olympus).

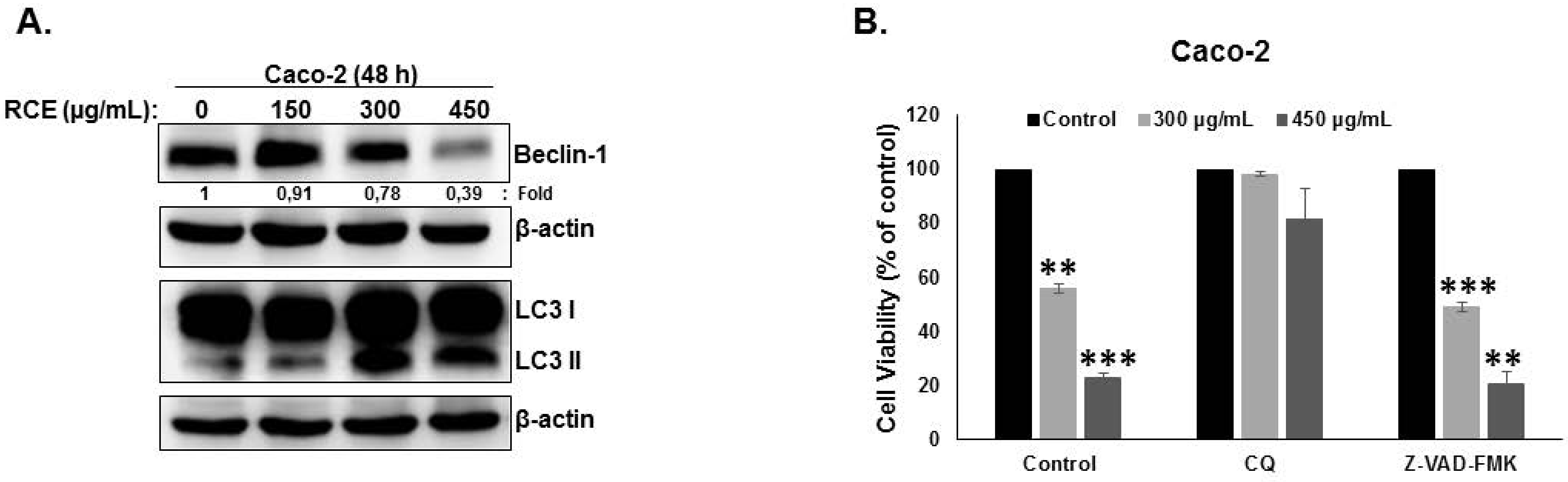

Figure S4. **Induction of Beclin-1-independent autophagy in Caco-2 cells by *Rhus coriaria*** (**A**) Western blotting analysis of LC3-II, and Beclin-1 expression in RCE-treated Caco-2 cells. Cells were treated with or without increasing concentrations of RCE for 48 h, then whole cell proteins were extracted and subjected to Western blot analysis, as described in Materials and Methods, for LC3-II, Beclin1 and β-actin (loading control) proteins. The western blots shown are representative of two independent experiments. (**B**) Inhibition of autophagy but not apoptosis reduces cell death induced by RCE in Caco-2 cells. Caco-2cells were pretreated with CQ or the pan-caspase inhibitor (Z-VAD-FMK) and then treated for 48 h with 300 or 450 µg/mL RCE. Cell viability was determined as described in Material and Methods using the Cell cytotoxicity assay kit (Abcam). Statistical analysis for cell viability data on control or treated cells were performed using one-way ANOVA followed by LSD Post-Hoc test to determine the significance (**$p < 0.005$, ***$p < 0.001$).

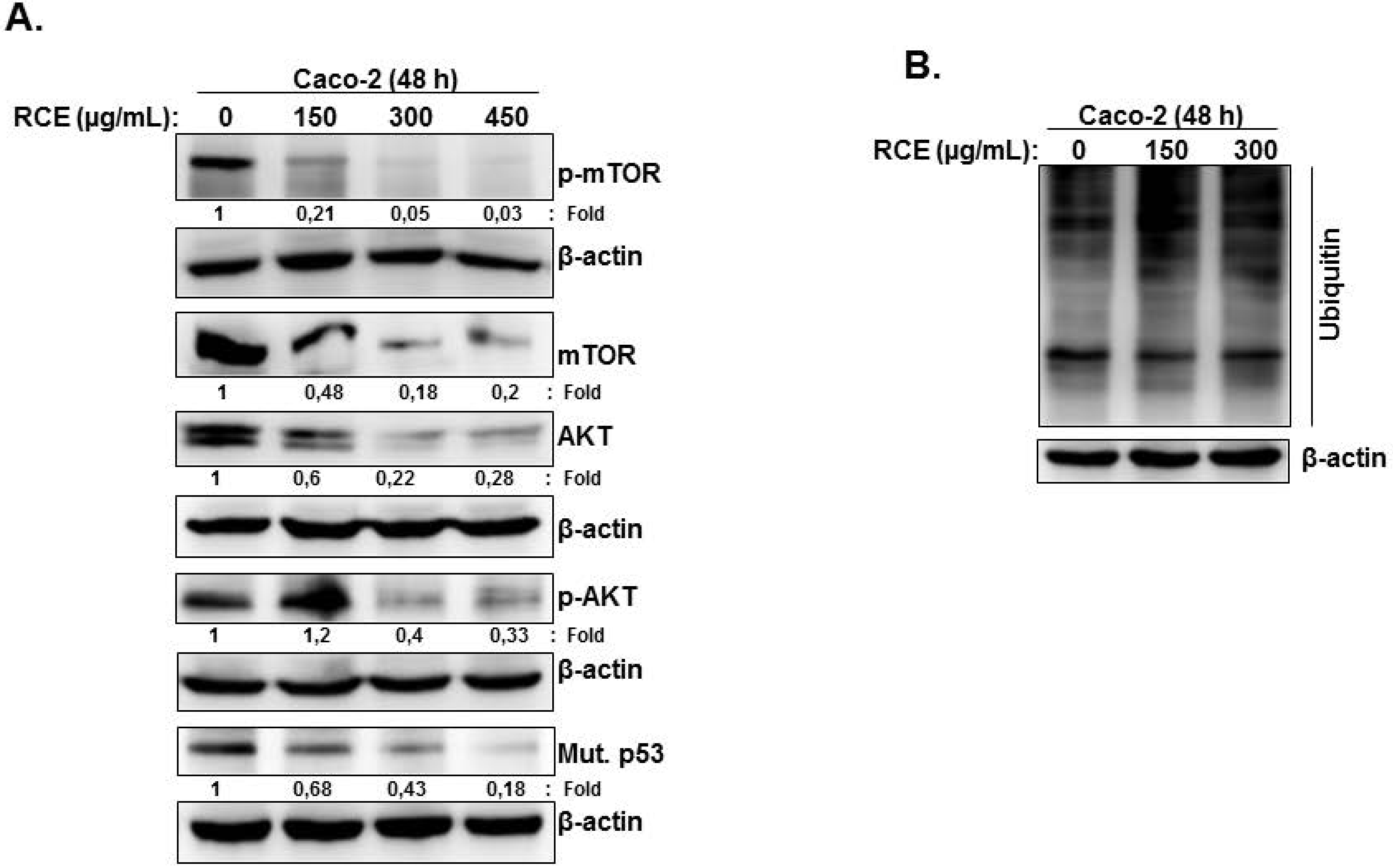

Figure S5. inhibition of the AKT/mTOR pathway, downregulation of p53 protein level and increase of the cellular level of protein ubiquitination in Caco-2 cells by Rhus coriaria. (**A**) Concentration-dependent decrease of phospho-mTOR, total mTOR, phospho-AKT,total AKT and p53 protein in RCE-treated Caco-2 cells. Cells were treated with or without increasing concentrations of RCE for 48 h, then whole-cell extracts were subjected to Western blot analysis. (**B**) RCE treatment increases the cellular level of ubiquitinated proteins in Caco-2 cells. Cells were treated with or without RCE (150 and 300 µg/mL) for 48 h, then whole-cell extracts were subjected to Western blot analysis for ubiquitinated proteins.

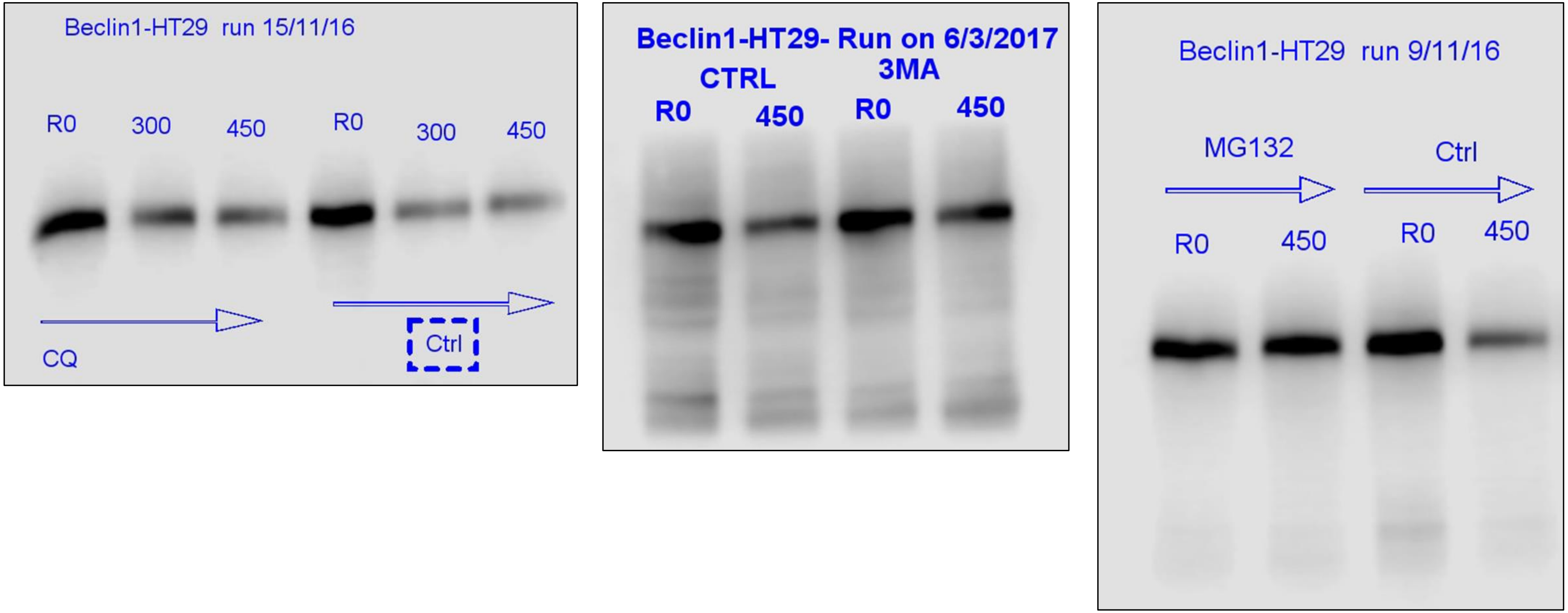

Figure S6: The full-length blots or original images for Figure 4 B, C and D.

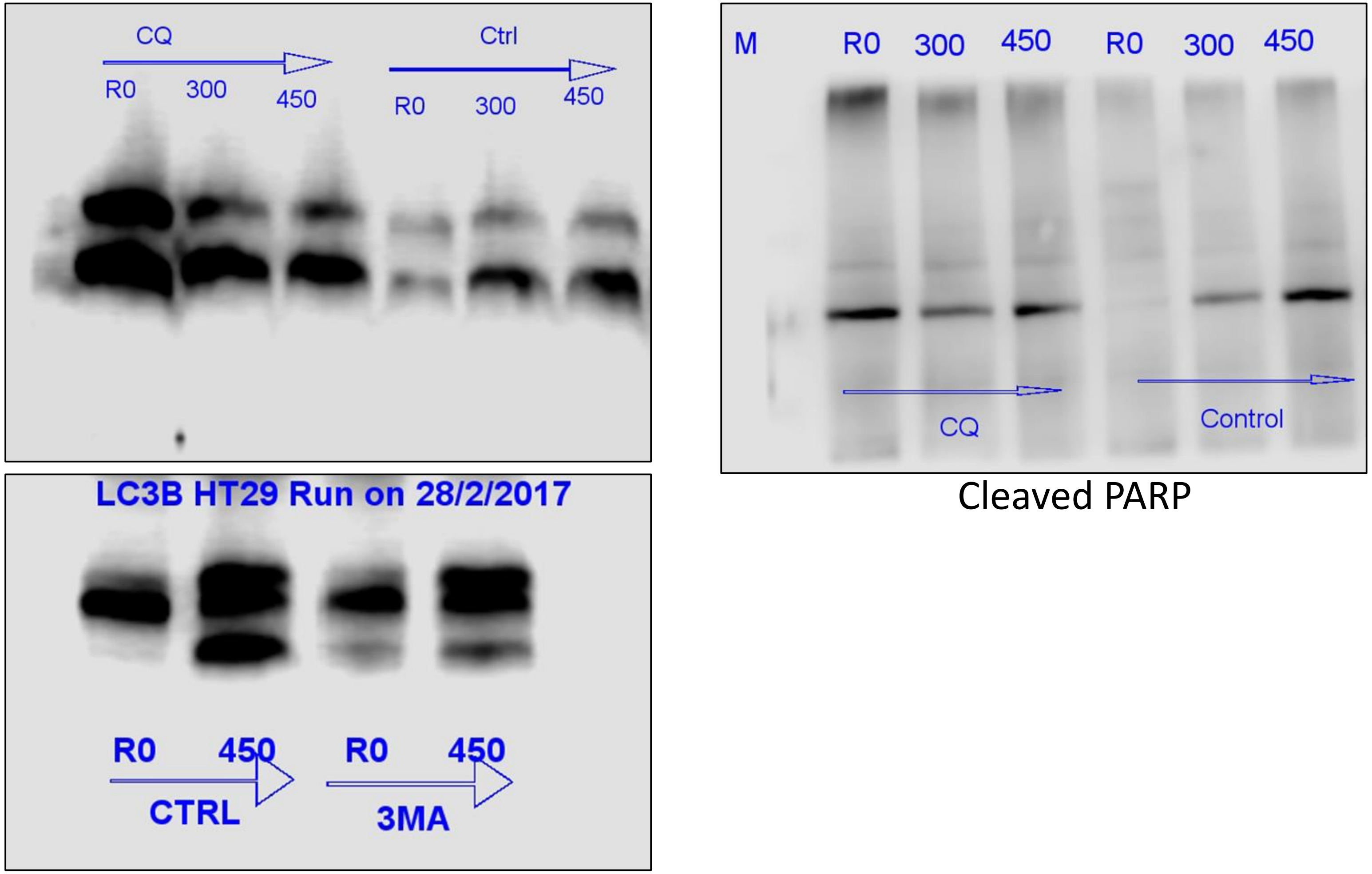

Figure S7: The full-length blots or original images for Figure 6B, C and F.

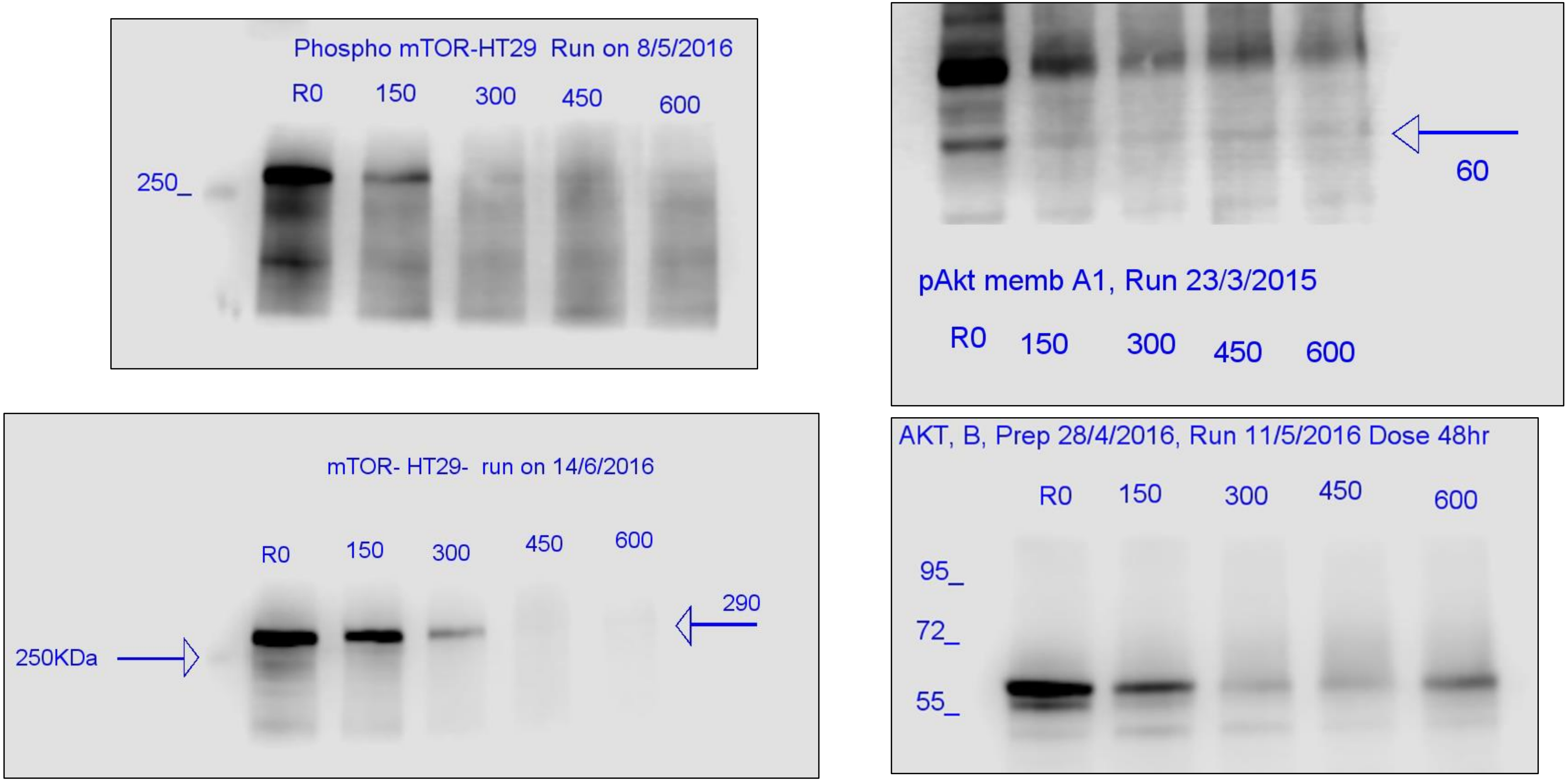

Figure S8: The full-length blots or original images for Figure 7A.

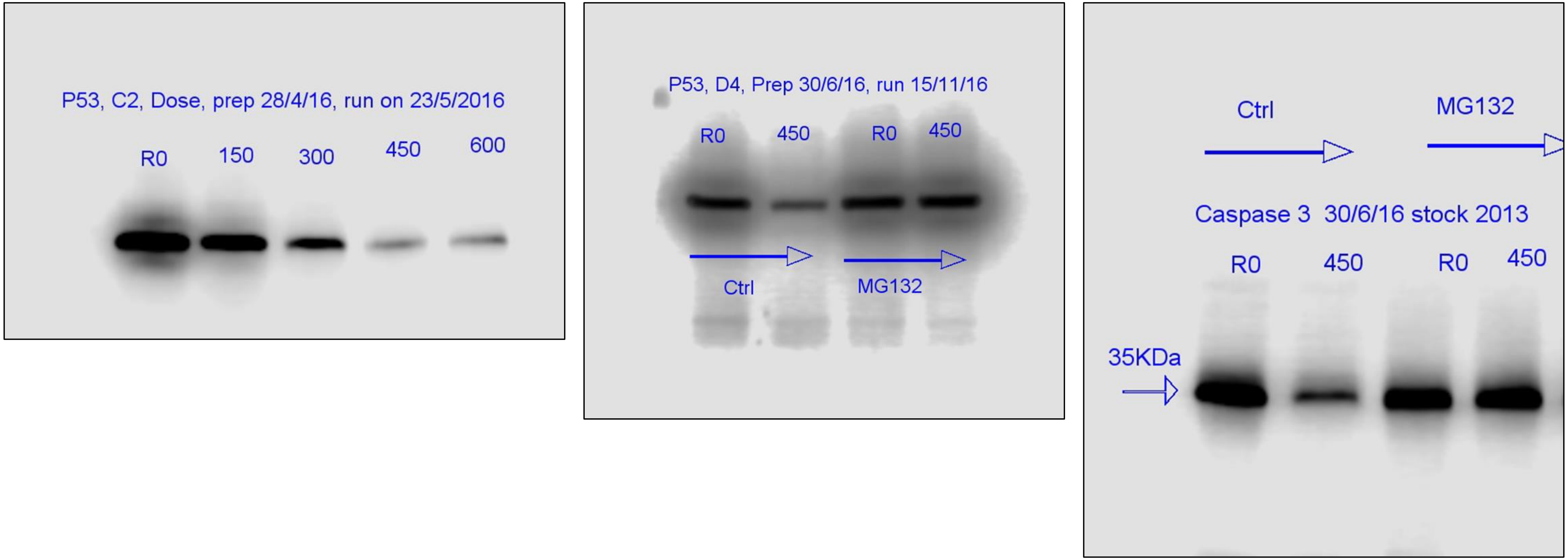

Figure S9: The full-length blots or original images for Figure 8A and C.

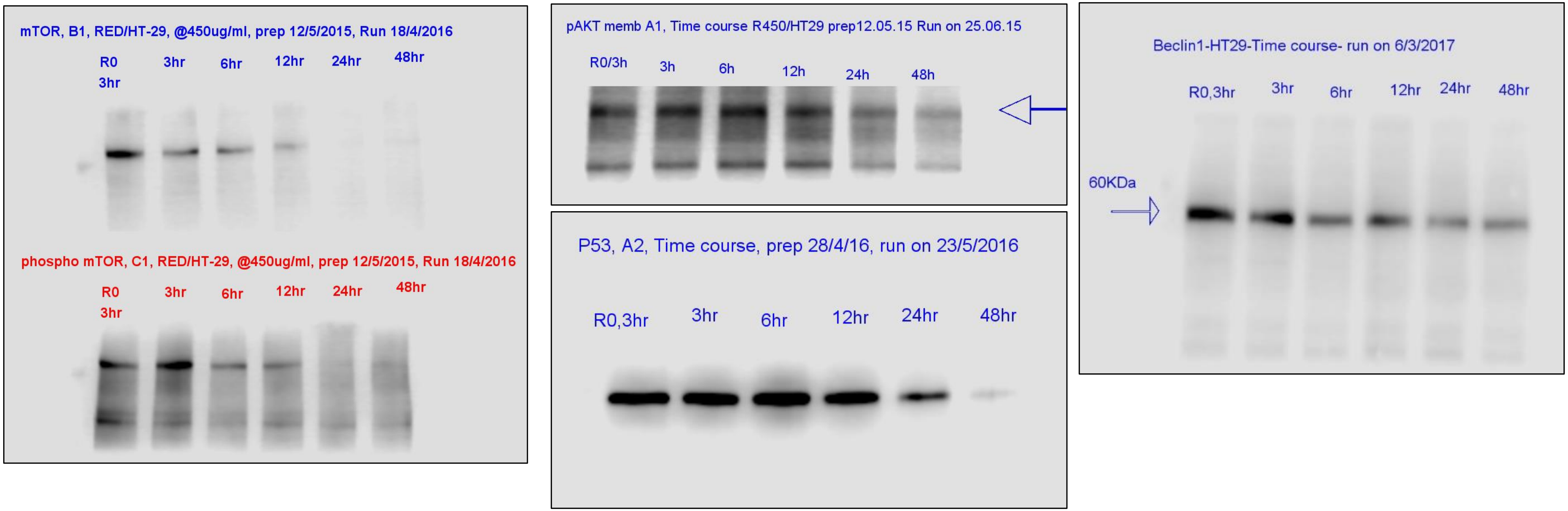

Figure S10: The full-length blots or original images for Figure 9A.
